# Effect of Remote Monitoring on Discharge to Home, Return to Activity, and Rehospitalization After Hip and Knee Arthroplasty: A Randomized Clinical Trial

**DOI:** 10.1001/jamanetworkopen.2020.28328

**Published:** 2020-12-21

**Authors:** Shivan J. Mehta, Eric Hume, Andrea B. Troxel, Catherine Reitz, Laurie Norton, Hannah Lacko, Caitlin McDonald, Jason Freeman, Noora Marcus, Kevin G. Volpp, David A. Asch

**Affiliations:** 1 Perelman School of Medicine, Department of Medicine, University of Pennsylvania, Philadelphia; 2 Center for Health Incentives and Behavioral Economics, University of Pennsylvania, Philadelphia; 3 Penn Medicine Center for Health Care Innovation, Philadelphia, Pennsylvania; 4 Department of Orthopaedic Surgery, University of Pennsylvania, Philadelphia; 5 Division of Biostatistics, Department of Population Health, NYU Grossman School of Medicine, New York, New York; 6 Center for Health Equity Research and Promotion, Philadelphia Veterans Affairs Medical Center, Philadelphia, Pennsylvania

## Abstract

**Importance:**

Hip and knee arthroplasty are the most common inpatient surgical procedures for Medicare beneficiaries in the US, with substantial variation in cost and quality. Whether remote monitoring incorporating insights from behavioral science might help improve outcomes and increase value of care remains unknown.

**Objective:**

To evaluate the effect of activity monitoring and bidirectional text messaging on the rate of discharge to home and clinical outcomes in patients receiving hip or knee arthroplasty.

**Design, Setting, and Participants:**

Randomized clinical trial conducted between February 7, 2018, and April 15, 2019. The setting was 2 urban hospitals at an academic health system. Participants were patients aged 18 to 85 years scheduled to undergo hip or knee arthroplasty with a Risk Assessment and Prediction Tool score of 6 to 8.

**Interventions:**

Eligible patients were randomized evenly to receive usual care (n = 153) or remote monitoring (n = 147). Those in the intervention arm who agreed received a wearable activity monitor to track step count, messaging about postoperative goals and milestones, pain score tracking, and connection to clinicians as needed. Patients assigned to receive monitoring were further randomized evenly to remote monitoring alone or remote monitoring with gamification and social support. Remote monitoring was offered before surgery, began at hospital discharge, and continued for 45 days postdischarge.

**Main Outcomes and Measures:**

The primary outcome was discharge status (home vs skilled nursing facility or inpatient rehabilitation). Prespecified secondary outcomes included change in average daily step count and rehospitalizations.

**Results:**

A total of 242 patients were analyzed (124 usual care, 118 intervention); median age was 66 years (interquartile range, 58-73 years); 78.1% were women, 45.5% were White, 43.4% were Black; and 81.4% in the intervention arm agreed to receive monitoring. There was no significant difference in the rate of discharge to home between the usual care arm (57.3%; 95% CI, 48.5%-65.9%) and the intervention arm (56.8%; 95% CI, 47.9%-65.7%) and no significant increase in step count in those receiving remote monitoring plus gamification and social support compared with remote monitoring alone. There was a statistically significant reduction in rehospitalization rate in the intervention arm (3.4%; 95% CI, 0.1%-6.7%) compared with the usual care arm (12.2%; 95% CI, 6.4%-18.0%) (*P* = .01).

**Conclusions and Relevance:**

In this study, the remote monitoring program did not increase rate of discharge to home after hip or knee arthroplasty, and gamification and social support did not increase activity levels. There was a significant reduction in rehospitalizations among those receiving the intervention, which may have resulted from goal setting and connection to the care team.

**Trial Registration:**

ClinicalTrials.gov Identifier: NCT03435549

## Key Points

**Question:**

Can a remote monitoring intervention that incorporates principles of behavioral science improve outcomes and value of care among patients undergoing hip and knee arthroplasty?

**Findings:**

In this randomized clinical trial of 242 patients, the remote monitoring program did not increase rate of discharge to home after hip and knee arthroplasty, and gamification and social support did not increase activity levels. However, a significant reduction in rehospitalizations among those assigned to the intervention was found.

**Meaning:**

In this study, remote monitoring did not increase discharge to home, but goal setting and connection to the care team may have reduced rehospitalizations.

## Teaser

This randomized clinical trial evaluates the effectiveness of activity monitoring and bidirectional text messaging on discharge to home and clinical outcomes after total hip arthroplasty or total knee arthroplasty among Medicare beneficiaries with intermediate risk of discharge to a facility.

## Introduction

Hip and knee replacements are the most common inpatient surgical procedures for Medicare beneficiaries in the US, with substantial cost and variability in care during the hospitalization and through postacute care.^1,2,3^ Most hospitals are participating in a bundled payment program for hip or knee replacement surgery, such as the comprehensive care for joint replacement model, with the goal of reducing unnecessary postacute care and rehospitalizations.^4,5,6^ Patients discharged from hospitals to facilities have higher costs and not necessarily improved outcomes, and payment policies have not resulted in substantially reduced rehospitalizations.^7,8,9,10^

Remote monitoring technologies such as activity monitoring and text messaging may help facilitate support for patients outside of traditional clinical settings, and could be used to help clinicians improve outcomes for this population in a scalable way.^11,12,13,14^ There is also an opportunity to integrate monitoring programs with the electronic health record, to ensure that this support occurs within the workflow and does not overburden clinicians.

Remote monitoring can also incorporate new insights from behavioral science to improve effectiveness and improve clinical care.^11,15^ Behavioral science has revealed that humans have predictable biases that might be harnessed to improve health promoting behavior.^16,17^ For example, framing discharge home as the safest option and providing recovery milestones might invoke social norms for clinicians, gamification and goal-setting could increase activity levels among patients postdischarge, and social support could improve patients’ adherence to clinical recommendations.^18,19,20^ Preliminary data showed that offering a remote monitoring program prior to hospitalization may encourage patients to elect to go home, and it may make clinicians feel more comfortable sending patients home.

In this pragmatic (conducted in routine practice) trial, we evaluated the effectiveness of offering activity monitoring and bidirectional text messaging on discharge to home and clinical outcomes after total hip arthroplasty (THA) or total knee arthroplasty (TKA) among patients with intermediate risk of discharge to a facility. We also evaluated whether gamification and social support increased activity levels for the patients receiving monitoring.

## Methods

### Study Design

This was a 2-arm pragmatic randomized clinical trial of usual care (arm 1) compared with remote monitoring (arm 2) to improve clinical outcomes and value of care after THA or TKA. Among those receiving remote activity monitoring, we also compared feedback alone (arm 2a) with feedback with gamification and social support (arm 2b) to evaluate whether activity levels increased . The study was approved by the Institutional Review Board at the University of Pennsylvania. A waiver of informed consent was obtained as the study was low risk and could not have practicably been carried out without the waiver. Having to obtain consent before enrolling in the study would have prevented the ability to evaluate whether offering the program had an effect on discharge to home. Patients did not receive compensation. The trial protocol and statistical analysis plan are available in Supplement 1. This study followed the Consolidated Standards of Reporting Trials (CONSORT) guideline for randomized clinical trials, including the diagram to track participants during enrollment and trial procedures (Figure).

**Figure.**
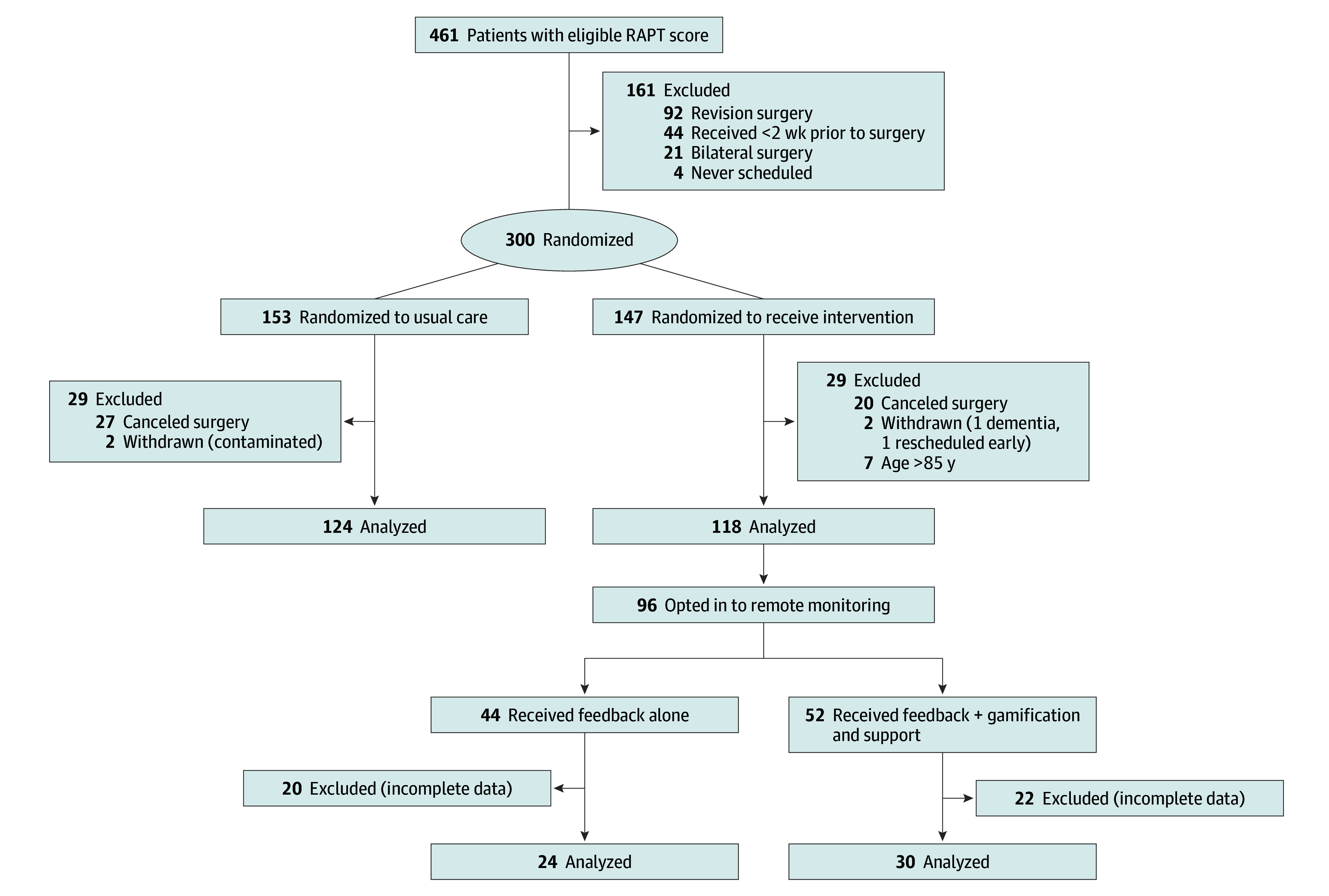
CONSORT Flow Diagram RAPT indicates Risk Assessment and Prediction Tool.

### Study Population

We included all patients between the ages of 18 and 85 years with a Risk Assessment and Prediction Tool (RAPT) score of 6 to 8 (of 12) who were scheduled to undergo THA or TKA at 2 hospitals in Philadelphia at the University of Pennsylvania.^21,22^ Patients with RAPT scores below 6 often require postdischarge facility care and those with scores above 9 can generally receive care at home.^23^ The range of 6 to 8 was chosen to reflect those patients with intermediate risk for whom the decision is unclear. We excluded patients if they had bilateral or revision surgery, dementia, end-stage kidney disease, cirrhosis, metastatic cancer, or another physical impairment (eg, amputation). Patients were recruited between February 7, 2018, and December 10, 2019.

### Recruitment and Randomization

Patients were asked to complete the RAPT survey as part of routine care during the initial surgical consultation or over the telephone with a surgical scheduler while making their appointment. Patients were then screened by research staff for eligibility criteria. Eligible patients were randomized in a 1:1 ratio in permuted blocks, stratified by hospital location and joint type, to either usual care (arm 1) or intervention (arm 2). Randomization and enrollment were accomplished via the Way to Health software platform (University of Pennsylvania), which facilitates and automates many aspects of study design and intervention implementation.^24^ Patients randomized to the intervention arm were further randomized in a 1:1 ratio to either remote monitoring (arm 2a) or remote monitoring with gamification and social support arm (arm 2b). Those in the intervention arm were invited to participate in activity monitoring by mailed invitation, which described the benefits of going home safely after surgery. Research staff followed up with up to 4 phone calls to discuss enrollment in the remote monitoring (HomeConnect+; University of Pennsylvania) program, and patients were given information about the benefits of remote monitoring with an activity monitor and text messaging. This outreach occurred prior to hospitalization for surgery (eFigure in Supplement 2).

### Interventions

Patients in the intervention arms who agreed received a physical activity monitor (Withings), daily pain score tracking through bidirectional text messaging, messaging about postsurgery milestones, nonadherence messaging, and access to clinicians as needed (eFigure in Supplement 2). Patients enrolled in arm 2b also received feedback with motivational messages using goal-setting and gamification, and identified a support partner to receive messaging. A message was sent via the electronic health record to each enrolled patient’s clinical care team, consisting of the surgeon, a nurse, and a social worker, notifying them of their patient’s inclusion in the remote monitoring program to encourage the patient to go home after surgery.

Patients in the intervention arms received an activity monitor, and if needed, a smartphone for texting and syncing their monitor to the Way to Health platform. These devices were either mailed to the patient for self-setup or set up in person after surgery at the hospital, depending on the patient’s comfort level with technology. Patients randomized and enrolled in arm 2b monitoring were also asked to identify a support partner for the duration of the program.

Remote monitoring and texting activities began at hospital discharge and were automated using the Way to Health platform. The intervention was offered to patients remotely in their homes or a facility after discharge. Patients in both intervention arms 2a and 2b received milestone messaging for recovery 1 to 3 times each week; message content was based on standard clinical materials given to patients. The activity tracker recorded daily step count and automatically transmitted step data to Way to Health. Participants were asked to report their pain score on a scale of 0 to 10 (0 = no pain, 10 = worst possible pain) each day for 2 weeks after discharge. If patients recorded a pain score greater than or equal to 7, they received messaging to contact the orthopedic surgery clinic if the pain was unmanageable (“Your pain score is high. If it feels unmanageable, call the Ortho hotline at [xxx-xxx-xxxx]”). If patients’ average step count decreased and mean pain score increased from the week before, study staff notified the patient’s clinical care team via an electronic health record message. Participants were called if their activity data had not synced for at least 3 days. All participants in the intervention arms received a follow-up survey through text link at the end of the program asking about their experience with the program as indicated in the trial protocol (Supplement 1).

Patients enrolled in study arm 2b also received feedback with motivational messages using goal-setting and gamification, and were asked to identify a support partner to receive messaging. For patients in this arm, the average step count in week 2 was considered baseline, and participants were encouraged to increase their step count by 5% every week to progress in reward levels. The levels at which patient can be rewarded are bronze, silver, gold, and platinum. All patients started at bronze at baseline. With appropriate progress, they moved in order to silver, gold and platinum levels. Social support partners for patients enrolled in this arm received messages if the participant did not upload activity data or submit pain scores for at least 4 days, asking the partner to reach out to the patient with support or encouragement. Content for all text messaging is included in Supplement 1. The investigators were masked to patient data and randomization, but the research staff were not masked because they were administering the intervention.

### Study Outcomes

The primary outcome was discharge status (home vs not home, eg, skilled nursing facility or in-patient rehabilitation facility). An additional primary outcome for patients receiving the intervention (those in arms 2a and 2b) was the mean change in daily steps from baseline (week 2) to the end of the intervention (week 6). Prespecified secondary outcomes included the number of days at home, Timed Up and Go (TUG) scores measured after surgery, rehospitalization rate, number of rehospitalizations, and emergency department visits.^25,26^ Rehospitalization rate is the percentage of patients rehospitalized at least once, and the number of rehospitalizations allows for multiple rehospitalizations per patient. Additional outcomes included patient satisfaction with the intervention (measured by postintervention text survey), outpatient visits, physical therapy visits, skilled nursing visits, occupational therapy visits, home health aide visits, and length of hospital stay.

### Statistical Analysis

Based on preliminary data from a pilot study, we expected that 53% of patients receiving usual care would be discharged to home. With a target enrollment of 300 patients, we estimated at least 80% power to detect an increase of 16 percentage points among those receiving the intervention (69% of patients discharged to home), using a 2-sided test with significance level set at *P* < .05. We compared discharge status using the χ^2^ test of proportions and intention-to-treat analysis. Among the 150 patients who were offered the intervention, we anticipated that approximately 70 patients would enroll in and receive remote monitoring, based on the pilot study. This number was based on the estimation that 60% of the patients randomized to the intervention would agree to receive monitoring and approximately 17% of patients in this population would not proceed with surgery. Assuming an SD in mean daily step count of 1200 with a 2-sided α of .05 based on the pilot results, we had 80% power to detect a difference between arms 2a and 2b in the increase in average daily step count from week 2 to week 6 of 800 steps. We used the independent group *t* test to compare the difference in mean daily step count from baseline to the end of the intervention between arms 2a and 2b. For secondary outcomes, we used the *t* test to compare differences in number of days at home, TUG score, number of rehospitalizations, and emergency department visits. We used the χ^2^ test of proportions to compare differences in rehospitalization rate. All analyses were performed using Stata statistical software version 16.0 (StataCorp LLC).

## Results

### Patient Characteristics

Three hundred patients were randomized and 242 patients were included in the main intent-to-treat analysis (124 usual care, 118 intervention); median age was 66 (interquartile range, 58-73); 78.1% (189) were women, 45.5% (110) were White, and 43.4% (105) were Black; and 66.9% (162) had knee arthroplasty (Table 1). Most patients (47 [16%]) not included in the analysis did not complete surgery because of cancellation or postponement unrelated to the study (Figure). Among those analyzed in the intervention arm, 81.4% (96) agreed to receive remote monitoring. There were no sociodemographic differences between those who agreed to receive monitoring compared with those who did not. The study intervention began with the first patient discharged from the hospital on February 23, 2018, and ended April 15, 2019, when the 45-day follow-up period ended for all randomized participants.

**Table 1.** Demographic Characteristics of Patients in the Main Analysis

Characteristic	No. (%)		
	Intervention	Control	Total
No.	118	124^a^	242
Age, median (IQR), y	66 (60-73)	66 (57-73)	66 (58-73)
Female sex	90 (76.3)	99 (80.0)	189 (78.1)
Race/ethnicity			
White	57 (48.3)	53 (42.7)	110 (45.5)
Black	47 (39.8)	58 (46.8)	105 (43.4)
Asian/Pacific Islander	3 (2.5)	2 (1.6)	5 (2.1)
Hispanic	2 (1.7)	1 (0.8)	3 (1.2)
Other/unknown	9 (7.6)	10 (8.1)	19 (7.8)
Annual income, median (IQR), $^b^	49 469 (32 741-73 231)	54 268 (32 741-75 328)	50 159 (32 741-74 114)
Surgery type			
Hip	40 (33.9)	40 (32.3)	80 (33.1)
Knee	78 (66.1)	84 (67.7)	162 (66.9)
Surgery location			
Hospital 1	89 (75.4)	92 (74.2)	181 (74.8)
Hospital 2	29 (24.6)	32 (25.8)	61 (25.2)
RAPT score, mean (SD)	7.2 (0.8)	7.2 (0.9)	7.2 (0.8)
Coverage type			
Commercial	28 (23.7)	22 (17.7)	50 (20.7)
Medicaid	15 (12.7)	18 (14.5)	33 (13.6)
Medicare	71 (60.2)	82 (66.1)	153 (63.2)
VA managed care	3 (2.5)	2 (1.6)	5 (2.1)
Worker’s compensation	1 (0.9)	0	1 (0.4)

Abbreviations: IQR, interquartile range; RAPT, Risk Assessment and Prediction Tool; VA, Veterans Affairs.

^a^ One patient died immediately after discharge and was censored from all analysis postdischarge (time at home, readmissions, emergency department visits, and outpatient visits).

^b^ Based on American Community Survey 2013-2017 5-Year Estimates Data.

### Clinical Outcomes

There was no difference in the rate of discharge to home between the usual care arm (57.3%; 95% CI, 48.5%-65.9%) and the intervention arm (56.8%; 95% CI, 47.9%-65.7%) (*P* = .95) (Table 2). There was a statistically significant reduction in rehospitalization rate in the intervention arm (3.4%; 95% CI, 0.1%-6.7%) compared with the usual care arm (12.2%; 95% CI, 6.4%-18.0%) (*P* = .01), as well as a reduction in the mean number of rehospitalizations (4.2 vs 13.0; *P* = .02). Among the 5 rehospitalizations in the intervention group, 1 was joint related (20%); among the 16 rehospitalizations in the control group, 7 were joint related (44%) (Table 2). There were no differences in length of hospital stay, number of days at home, number of office visits, number of emergency department visits, or TUG scores postsurgery between arms (eTable 1 in Supplement 2).

**Table 2.** Hospitalization/Discharge and Use Data

Variable	Intervention (n = 118)	Control (n = 124)^a^	*P* value
Discharge to home, No. (%) [95% CI]	67 (56.8) [47.9-65.7]	71 (57.3) [48.5-65.9]	.95
Length of hospital stay, mean (SD), d	2.5 (1.0)	2.5 (1.3)	.96
Time at home, median (IQR), d^b^	42 (34-43)	42 (33-43)	.64
Rehospitalization rate, No./total No. (%) [95% CI]	4/118 (3.4) [0.1-6.7]	15/123 (12.2) [6.4-18.0]	.01
Rehospitalizations, total No. (%) [95% CI]	5 (4.2) [0.6-7.9]	16 (13.0) [7.1-19.0]	.02
Observation^c^	1 (0.8)	1 (0.8)	NA
Inpatient^c^	4 (3.4)	13 (10.5)	
Patients with 2 rehospitalizations	1 (0.8)	1 (0.8)	
Admissions from ED	2 (1.7)	9 (7.3)	
Joint-related rehospitalizations	1 (0.8)	7 (5.6)	
Rehospitalizations by location, No. (%)			
University of Pennsylvania	1 (0.8)	13 (10.5)	
Outside hospital			
By care everywhere	4 (3.4)	1 (0.8)	
By patient report	0	2 (1.6)	
Days to first rehospitalization postdischarge, median (IQR)	8 (7-14)	20 (8-33)	.19
ED visit rate, No./total No. (%) [95% CI]	6/118 (5.1) [1.1-9.1]	14/123 (11.4) [5.8-17.0]	.08
ED visits, total No. (%) [95% CI]	7 (5.9) [1.7-10.2]	16 (13.0) [7.1-19.0]	.06
Patients with 1 ED visit	5 (4.2)	12 (9.7)	NA
Patients with 2 ED visits	1 (0.8)	2 (1.6)	
Not admitted to hospital	5 (4.2)	7 (5.6)	
Office visits, No., mean (SD)	1.6 (1.1)	1.6 (0.9)	.52
Physical therapy visits, No., mean (SD)	5.3 (4.1)	5.4 (4.2)	.82
Skilled nursing visits, No., mean (SD)	3.4 (2.9)	3.6 (3.0)	.53
Occupational therapy visits, No., mean (SD)	1.6 (2.0)	1.9 (2.2)	.22
Home health aide visits, No., mean (SD)	0.03 (0.2)	0.04 (0.5)	.88

Abbreviations: ED, emergency department; IQR, interquartile range; NA, not applicable.

^a^ One patient died immediately following discharge and was censored from all analysis postdischarge.

^b^ Patients with missing facility discharge data are censored.

^c^ Rehospitalization class is missing for hospitalizations outside of Pennsylvania.

### Step Analysis

Ninety-six patients (81.4%) agreed to receive the remote monitoring program; 44 (45.8%) patients were randomized to receive feedback alone (arm 2a) and 52 (54.2%) to receive feedback plus gamification and social support (arm 2b) (Table 3). The median age of these participants was 68 years (interquartile range, 61-74 years); they were primarily women (75.0%), White (51.0%), and receiving knee replacement surgery (66.7%).

**Table 3.** Demographic Characteristics of Patients in Step Analysis

Characteristic	No. (%)		Total
	2a (Remote monitoring)	2b (Remote monitoring + gamification & social support)	
No.	44	52	96
Age, median (IQR), y	68 (64-74)	66 (60-74)	68 (61-74)
Female sex	31 (70.5)	41 (78.8)	72 (75.0)
Race/ethnicity			
White	23 (52.3)	26 (50.0)	49 (51.0)
Black	14 (31.8)	20 (38.5)	34 (35.4)
Asian/Pacific Islander	1 (2.3)	2 (3.8)	3 (3.1)
Hispanic	0	2 (3.8)	2 (2.1)
Other/unknown	6 (13.6)	2 (3.8)	8 (8.3)
Annual income, median (IQR), $^a^	51 465 (32 741-82 081)	48 125 (29 581-71 248)	49 469 (32 741-78 165)
Surgery type			
Hip	18 (40.9)	14 (26.9)	32 (33.3)
Knee	26 (59.1)	38 (73.1)	64 (66.7)
Surgery location			
Hospital 1	33 (75)	40 (76.9)	73 (76)
Hospital 2	11 (25)	12 (23.1)	23 (24)
RAPT score, mean (SD)	7.3 (0.8)	7.2 (0.8)	7.2 (0.8)
Coverage type			
Commercial	7 (15.9)	14 (26.9)	21 (21.9)
Medicaid	4 (9.1)	6 (11.5)	10 (10.4)
Medicare	30 (68.2)	31 (59.6)	61 (63.5)
VA managed care	2 (4.5)	1 (1.9)	3 (3.1)
Worker’s compensation	1 (2.3)	0	1 (1)

Abbreviations: IQR, interquartile range; RAPT, Risk Assessment and Prediction Tool; VA, Veterans Affairs.

^a^ Based on American Community Survey 2013-2017 5-Year Estimates Data.

There was no difference in the amount of data uploaded between groups (eTable 2 in Supplement 2); 54 (56.3%) patients submitted data each week between baseline (week 2) and end of study (week 6). There was a mean (SD) increase in daily step count of 833 (1303) in both groups combined from week 2 to week 6, but there was no significant difference between the gamification and social support arm (arm 2b) compared with feedback alone (arm 2a) (Table 4).

**Table 4.** Change in Mean Daily Step Count From Week 2 to Week 6

Study arm	No.	Steps per day, mean (SD)		Step increase, mean (SD) [95% CI]	*P* value^a^
		Week 2	Week 6		
2a^b^	24	931 (785)	1561 (1766)	630 (1372) [50.9-1209.5]	.31
2b^c^	30	916 (697)	1911 (1473)	995 (1244) [530.3-1459.5]	
Combined	54	923 (730)	1756 (1604)	833 (1303) [477.2-1188.4]	NA

^a^ Independent group *t* test.

^b^ Arm 2a consisted of patients receiving remote activity monitoring, feedback alone.

^c^ Arm 2b consisted of patients receiving remote activity monitoring, feedback with gamification and social support.

### Postintervention Survey

Of the 96 patients receiving monitoring, 55 (57%) completed the postintervention survey. On a scale of 1 to 10 (1 = extremely unlikely, 10 = extremely likely), participants expressed a mean (SD) score of 8.8 (2.1) in describing the likelihood of recommending the remote monitoring program to other patients undergoing joint replacement surgery and 85% reported a score of 8 or higher. Participants also agreed that the program made them feel more connected to the care team (71% strongly agreed or agreed) and more comfortable going home (64% strongly agreed or agreed).

## Discussion

In this pragmatic randomized clinical trial, we found that the activity monitoring and text messaging program did not increase the rate of discharge to home after hip and knee arthroplasty, but was associated with a reduction in rehospitalizations. Activity levels were modest after hospital discharge, and gamification with social support did not significantly increase step count.

There are a few reasons why offering the intervention was not sufficient to increase discharge to home, despite a small pilot study suggesting potential benefit. First, the patients included in this trial were patients at intermediate risk with a RAPT score between 6 and 8, so they may have had physical or social barriers to going home that remote monitoring could not address. While RAPT score accurately estimates discharge disposition for patients at high and low risk, the evidence is limited about patients at intermediate risk.^23^ Second, the decision about discharge disposition involves patients, family members, and an interdisciplinary team of clinicians and staff. Our intervention was low touch and introduced in the outpatient setting to patients, while the decision to discharge home is largely associated with the inpatient clinical team through discussions with the patient. Third, there were already other efforts to increase discharge to home at the participating hospitals, such as home physical therapy and counseling. In this pragmatic trial, we evaluated only the monitoring program in addition to usual care. Fourth, 81.4% of the patients agreed to receive monitoring, and many did not consistently use the activity monitors. In our intention-to-treat analysis, we included all patients who were randomized regardless of motivation to participate. Notably, 78.1% of the patients in the trial were women, who may have had a differential response to the intervention.

Another notable finding from this trial was that those assigned to the intervention arm were less likely to return to the hospital after discharge. There are a few potential mechanisms for these findings. First, the intervention provided feedback about pain score, as well as instructions to call the practice in case of issues. This engagement may have redirected the patients from calling their primary care physician or going to the local emergency department for care that could have resulted in a hospitalization. Patients were given a direct telephone line to evaluate any urgent issues without having to go through conventional communication channels. This approach is supported by data showing that 20% of rehospitalizations were joint related in the intervention group compared with 44% in the control group. Second, the text messaging included content about milestones and behaviors that the patients should engage in, such as hip or knee exercises, physical activity, and medication management. Although the same instructions are communicated by the surgery team, the text messaging may have provided reinforcement in real-time to improve adherence and accountability. Similarly, activity monitoring might have encouraged increases in step count, which may have improved recovery function. Prior studies describing multimodal comprehensive programs reported a reduction in readmissions but were limited by observational pre-post designs.^10,27,28^ A randomized clinical trial of text messaging reported increased activity levels but did show a statistically significant effect on emergency department use.^13^

To our knowledge, this study is also 1 of the largest to evaluate activity levels among patients undergoing THA and TKA after discharge using wearable devices. We found that gamification and social support did not significantly improve step count, despite prior studies showing effectiveness. Our results may differ for several reasons. First, step counts and increases were modest in the 6 weeks after hospital discharge, so this population may not have had the functional capacity to achieve substantial increases in activity.^29^ Second, many participants struggled with getting the activity monitors and smartphone application to sync with the device. Third, the gamification was mainly gain-framed, and there could have been more effect from stronger loss-framed gamification, competition, or larger step goals.^19,30^ Prospect theory suggests that people respond asymmetrically to loss and gain perspectives.^31^

### Strengths and Limitations

This study has strengths, including its prospective design with patient-level randomization. It was pragmatic in including all patients with an eligible RAPT score, and the waiver of informed consent allowed an examination of the kind of unrestricted patient population this program would apply to in routine clinical practice, maximizing potential generalizability to other populations. The study population was diverse, situated in 2 urban hospitals, and included 43% Black patients, who have had higher rates of adverse outcomes after the procedure.^32^

This study had limitations. Although it did not demonstrate effectiveness against the primary outcome, discharge to home, it showed effectiveness against a prespecified secondary outcome, rehospitalization. The intervention itself was multimodal and whether specific elements of this compound intervention might be responsible for the favorable result is not clear. Future studies could specifically evaluate usability of technology and its association with clinical outcomes and adherence.^33,34^

## Conclusions

This pragmatic randomized clinical trial suggests that remote monitoring could enhance care for patients after THA and TKA. While the rate of discharge to home was not increased, there was high engagement in the intervention and a reduced rate of rehospitalizations. Future work is warranted to help determine which aspects of the intervention were effective and how these types of remote monitoring approaches could be applied to other postsurgery populations.

## Notes

Supplement 1.
Trial Protocol

Supplement 2. **eTable 1.** Timed Up and Go (TUG) Scores **eTable 2.** Device Usage by Study Arm (n=96, All pts Who Opted to Receive Device, Weeks 1-6) **eFigure.** Intervention Flow Diagram

Supplement 3.
Data Sharing Statement
