# Errors in SDs in Results and Table 4

**DOI:** 10.1001/jamanetworkopen.2024.12345

**Published:** 2024-04-17

In the Original Investigation titled “Effect of Remote Monitoring on Discharge to Home, Return to Activity, and Rehospitalization After Hip and Knee Arthroplasty: A Randomized Clinical Trial,”^1^ published December 21, 2020, there were errors in the presentation of SDs. For the mean (SD) given in Results as “833 (177),” the SD should have been “1303.” In Table 4, the SD value given as “280” should have been “1372,” the value given as “227” should have been “1244,” and the value given as “177” should have been “1303.” This article was corrected.^1^
