# Supplementary material for: Effect of Remote Monitoring on Discharge to Home, Return to Activity, and Rehospitalization After Hip and Knee Arthroplasty: A Randomized Clinical Trial
Source: JAMA Netw Open. 2020 Dec 21;3(12):e2028328. doi: 10.1001/jamanetworkopen.2020.28328 (PMC7753899; doi:10.1001/jamanetworkopen.2020.28328)
Supplement: Supplement 1. — Trial Protocol [file jamanetwopen-e2028328-s001.pdf]

Supplement

Automated Hovering for Joint Replacement Surgery

This supplement provides additional information about the work. It contains the following items:

Initial Protocol ………. 1

Final Protocol …… 12

Summary of protocol changes ……………………………………………………………………………………………….. 24

Original statistical analysis plan …………………………………………………………………………………………… 26

Final statistical analysis plan ………………………………………………………………………………………………….. 27

Summary of statistical analysis plan changes ………………………………………………………………………….28

Appendix A: Text Messaging Content ………………………………………………………………………………………29

Appendix B: End of Study Final Survey Instrument…………………………………………………………………….32

Initial Protocol

Abstract
The goal of this study is to test the approach of automated hovering to encourage patients to be discharged to home safely after lower extremity joint replacement surgery. Among UPHS patients, we will target those undergoing hip or knee replacement surgery at PPMC and PAH. Our aim is to increase the number of patients that are discharged to home, by providing patients with a physical activity pedometer, daily pain score tracking through bi-directional text messaging, milestone and nudge messaging for recovery, social influence, and connection to clinicians as needed for 6 weeks after surgery. Eligible patients are age 18-85, with a Risk Assessment and Prediction Tool (RAPT) score of 6-8 and are scheduled to undergo lower-extremity joint replacement surgery. Patients will be randomized 1:1 to usual care (Arm 1) and intervention (Arms 2a and 2b). After enrollment with the monitoring device, patients in the intervention group will be randomized 1:1 to Arm 2a remote monitoring or Arm 2b remote monitoring + goal-setting and social support). Both intervention arms will receive the intervention for 6 weeks post-surgery.

Study Instruments
All subjects enrolled in either Arm 2a or 2b will receive a follow-up survey at the end of the program asking about their experience with HomeConnect+.

Group Modifications
Only participants enrolled in one of the intervention arms (2a or 2b) will receive an activity monitor, daily monitoring, milestone messaging and connection to clinicians as needed for 6 weeks after their surgery. Participants enrolled in Arm 2b will additionally receive nudge messaging to increase step count, feedback with motivational messages of patient progress, and social nudges from a support partner.

Administration of Surveys
All subjects enrolled in either Arm 2a or 2b will receive a follow-up survey at the end of the program asking about their experience with HomeConnect+. This survey will be accessible via a text link and will take approximately 5 minutes to complete.

Objectives
<u>1.1 Objectives</u>
The specific aims of this study are to: Aim 1: provide remote monitoring with nudges to patients, support partner, and staff to encourage patient discharge to home. Aim 2: Obtain additional data about the implications of activity monitoring data and clinical outcomes.

<u>1.2 Primary Outcome Variable</u>
The primary outcome will be the percent of patients that are discharged to home. An additional primary outcome for intervention patients is the percent step increase.

<u>1.3 Secondary Outcome Variable(s)</u>
The secondary outcomes will be days at home, TUG score, patient reported outcomes, patient experience, readmissions, overall utilization, length of stay, time to home, increase in average daily step count from week 2 to week.

Background

<u>1.1 Program Goals</u>

Hip and knee replacements are the most common inpatient surgery for Medicare beneficiaries, with substantial cost during the hospitalization and through post-acute care, where there is substantial variability. The University of Pennsylvania Health System (UPHS) participates in voluntary Medicare, IBC, and Horizon bundled payment programs, in addition to being subject to readmission penalties by Medicare. UPHS discharges about 70% of patients to home (53% of patients with RAPT score 6-8), and patients may not engage in the recommended physical therapy activities to achieve optimal clinical outcomes. For this patient population, alternatives to discharge to home are inpatient rehab facility and a Skilled Nursing Facility or SNF. Patients that are discharged to inpatient rehab or SNF are more likely to have post-discharge severe adverse events and unplanned readmissions than patients that are discharged to home post-surgery.

During the last year, our team conducted contextual inquiry and rapid cycle experimentation with the goal of improving the value and outcomes for patients who received joint replacement surgery. This work was conducted as part of a QI initiative with the Penn Medicine MSK service line. We first conducted chart review of discharge disposition by RAPT score for LEJR patients in 2016. We correlated the RAPT score with patient intention and their ultimate discharge destination. Then, we conducted observations and interviews of patients and Orthopedics Care Team members about discharge disposition decision process. The goal was to better understand the existing processes to identify areas for improvement. From this work we learned that patient preference was important to disposition and home was not necessarily offered as the preferred option before surgery.

From shadowing patients pre-op appointments with Social Work as they discussed disposition goals with LEJR patients we discovered that Social Work can influence pre-op goal setting. We also discovered that offering home as a default choice (instead of an active choice) for patients with RAPT score 9 (low risk) will increase the number of patients discharged to home (from 68% to 73%). In addition, we learned that by giving physical therapists information about step counts and daily pain scores, did not increase their confidence in discharging patients from home PT sooner. As part of the rapid cycle experimentation we provided remote monitoring and feedback via text messaging in a program called HomeConnect Pilot to patients undergoing LEJR surgery with RAPT score 6-8 from March to June 2017. The HomeConnect Pilot, offered by the Orthopedics Department at Penn Medicine, was designed to help keep the patient connected with their care team after surgery through the use of a remote monitoring devices (pedometer) and text messaging, such as daily pain score. The HomeConnect program lasted for 30 days after surgery and was offered as a voluntary program in addition to the patient's usual care. We obtained verbal consent from patients for text messaging as guided by the Privacy Officer of Penn Medicine, as has been done for other text messaging interventions. In this pilot, 14 medium-risk patients (RAPT 6-8) were enrolled in Way to Health before surgery and showed the feasibility and acceptability of automated hovering with 73% using the devices with robust data about daily activity and pain scores. 79% of patients enrolled in the platform went home, which was higher than the baseline rate of 53% for medium-risk patients (RAPT 6-8). Through this pilot we also discovered that the LEJR patient population can safely use the pedometer. We also discovered that collecting daily step count and daily text messaging with this population is feasible. Monitoring of daily step count and pain score for the patients enrolled in the HomeConnect Pilot was done through the Way to Health platform. The Way to Health platform is a PennMedicine platform, hosted on site at the University of Pennsylvania and is protected by a secure firewall. All patient information was stored in EPIC, RedCap, or Way to Health, all HIPAA compliant platforms. For the purposes of dissemination, no patient identifiers will be used and data will be provided in the aggregate.

Statistical Considerations

1.1 Power and sample size

We expect the rate of discharge to home to be approximately 53% through usual care. With 300 patients we will have at least 80% power to detect an increase of 15% among those receiving the intervention, using a two-sided test with a significance level of 0.05. We anticipate approximately 35 patients in arms 2a and 2b. Based on a standard deviation in average daily step count of 1200 with a two sided alpha of 0.05, we have 80% power to detect an increase in average daily step count of 800 from week 2 to week 6 in arm 2b as compared to arm 2a.

1.2 Data analysis

We expect the rate of discharge to home to be approximately 53% through usual care. With 300 patients we will have at least 80% power to detect an increase of 15% among those receiving the intervention, using a two-sided test with a significance level of 0.05. We anticipate approximately 35 patients in arms 2a and 2b. Based on a standard deviation in average daily step count of 1200 with a two sided alpha of 0.05, we have 80% power to detect an increase in average daily step count of 800 from week 2 to week 6 in arm 2b as compared to arm 2a.

Study Design

1.1 Design

In this study, the Orthopedic surgery scheduler will help the clinical research coordinator (CRC) identify appropriate patients who are planning inpatient joint replacement at UPHS. Patients will be randomized 1:1 to usual care (Arm 1) and intervention (Arms 2a and 2b). Patients randomized to the intervention will be randomized 1:1 to Arm 2a remote monitoring or Arm 2b remote monitoring + goalsetting and social support. Both intervention arms will receive the intervention for 6 weeks post-surgery.

Randomization will be stratified by hospital (PPMC vs PAH), and surgery type (hip vs. knee). Patients will be randomized via the Way to Health platform prior to outreach. Patients will be randomized prior to outreach with a waiver of consent for the following reasons: 1) the study is low risk 2) the patients randomized to Arm 1 (usual care) receive standard and routine clinical care, and this study does not violate their welfare, 3) the consent process itself may affect the outcome of our study.

To further explain number 3, patients that are randomized to Arms 2a and 2b (automated hovering) will be invited to join our study by mail and phone. These patients will be explained the benefits of going home after surgery and the ways in which we will provide (automated) support for the patient when they are home after surgery. Since the explanation of our study encourages patients to go home after surgery (our primary outcome), and we are not interacting with all patients, a waiver of consent is needed. Patients randomized to Arms 2a and 2b (intervention arms) will still have to opt-in to the intervention. The enrolled patients in Arms 2a and 2b will receive a physical activity monitor, daily pain score tracking through bi-directional text messaging, milestone messaging, non-adherence messaging, and connection to clinicians as need. Patients enrolled in Arm 2b, remote monitoring + goal-setting and social support, will also receive nudge messaging to increase step count, feedback with motivational messages of patient progress, and social nudges from a support partner. The patients in Arms 2a and 2b will receive the automated feedback for 6 weeks post-surgery. A detailed outline of study design can be found in the procedures sections.

1.2 Consent Process

This study is minimal risk as all patients are receiving standard clinical care. This study was designed in collaboration with the Penn Medicine Musculoskeletal Service Line. The intervention is clinically appropriate and aligns with the standard patient clinical care, and the consent process itself may influence the outcome of our study. The purpose of the HomeConnect+ program is to encourage patients to go home after surgery. The invitation to be part of HomeConnect+ includes language to nudge the patient to choose home as their discharge goal. Explaining the program to a potential participant randomized to the Usual Care arm may influence the patients decision making process for discharge disposition. It would be impractical to obtain a written signature for consent and HIPPA authorization. We are requesting a waiver of the requirement to document consent and HIPPA authorization with signature for participants enrolled in the intervention since we believe the research presents no more than minimal risk of harm to subjects and involves no procedures for which written consent is normally required outside of the research context. Participation in the intervention is completely voluntary. Participants randomized to the intervention arms still need to opt-in to the study. All participants receive standard clinical care. Participants randomized to the intervention arms will be informed that this program is voluntary and that they can stop participating in the program (stop using the activity pedometer) at any time.

Study Duration

The duration of participation for the intervention will be 6 weeks after discharge from the hospital. We expect the total duration of the study to last 8 months for all patients to complete the intervention, however time from schedule to surgery is highly variable for LEJR. We will recruit patients from December 2017 through June 2018. We estimate that the study will be completed by September 2018.

Resources Necessary for Human Research Protections

The project will take place at the Leonard Davis Institute Center for Health Incentives and Behavioral Economics (LDI CHIBE) at the University of Pennsylvania (UPENN). The team includes investigators experienced in clinical medicine, health behavior interventions, clinical trials, behavioral economics, cost-effectiveness analysis, and program evaluation. Our partnership combines the resources and capabilities of a major university (the Wharton School and the Perelman School of Medicine at the University of Pennsylvania). This study will have the support of multiple PIs: Dr. Shivan Mehta, MD, MBA, MSHP, is the Director of Operations for the Penn Medicine Center for Health Care Innovation. Dr. Kevin Volpp, MD, PhD, directs the LDI CHIBE and the NIA-funded PENN-CMU Roybal P30 Center on Behavioral Economics and Health and is a Professor of Medicine at the Perelman School of Medicine (SOM) and Professor of Health Care Management at the Wharton School at UPENN. David Asch, MD, MBA - is Executive Director of the Penn Medicine Center for Innovation, Professor of Health Care Management and Economics and Professor of Operations and Information Management at Wharton and Professor of Medicine at Perelman. This study will be supported on a secure web portal on the Way to Health platform, modified to the specifications of this study.

Target Population

Eligibility Criteria: UPHS patients between the ages of 18-85 with a Risk Assessment and Prediction Tool (RAPT) of 6-8 and scheduled to undergo lower-extremity joint replacement surgery. The total target enrollment will be 300 patients. 150 in usual care (Arm 1) and 150 in the intervention arms (Arm 2a and 2b). We estimate that roughly 70 participants in the intervention arms (35 per arm) will be active in this study. This number is based on the estimation that 60% of the patients randomized to the intervention will opt-in to the study and roughly 17% of patients in this population do not proceed with surgery.

Subjects Enrolled by Penn Researchers
300

Subjects Enrolled by Collaborating Researchers
0

Accrual
Participants in this study will be undergoing LEJR surgery at Penn Medicine. They will be identified by the Orthopedic surgery schedulers and shared with our study staff. A study coordinator will review the list of patients to determine eligibility. If a patient meets the minimal requirements they will be entered into the Way to Health platform for randomization. The study coordinator will send a mailed invitation and brochure followed by reaching out via phone to the patients randomized to Arm 2a (remote monitoring) or Arm 3 (remote monitoring + goal -setting and social support) to invite these patients to partake in our study.

Key Inclusion Criteria
Eligibility Criteria: UPHS patients between the ages of 18-85 with a Risk Assessment and Prediction Tool (RAPT) of 6-8 and scheduled to undergo primary lower-extremity joint replacement surgery. Patients that enroll in the HomeConnect program and are discharge to a SNF can still be part of the HomeConnect program; we will still track their daily step count and pain score. Patients with a RAPT score of 6-8 are considered low risk and able to go home after surgery, however there are things that may occur in the patients care post-surgery that may change their discharge disposition to SNF or an inpatient rehab facility. For this reason, we have included days at home and length of stay as part of our secondary outcomes.

Key Exclusion Criteria
Exclusion Criteria: Patients will be excluded if they do not meet all of the inclusion criteria, or if they have bilateral or revision surgery, dementia, end stage renal disease, cirrhosis, metastatic cancer, non-English speaker, or other physical impairment (ex. amputation).

Vulnerable Populations
No vulnerable Populations are included in the research study.

Populations Vulnerable to Undue Influence or Coercion
We are not specifically targeting any vulnerable populations.

Subject Recruitment
The Orthopedic surgery scheduler will help the clinical research coordinator (CRC) identify appropriate patients who are planning inpatient joint replacement at UPHS. Patients will be randomized 1:1 to usual care (Arm 1) and intervention (Arm 2a remote monitoring and Arm 2b remote monitoring + goalsetting and social support). If a patient meets the minimal requirements they will be entered into the Way to Health platform for randomization prior to outreach. The study coordinator will send a mailed invitation and then reach out via phone or in-person recruitment (up to 4 phone calls) to the patients randomized to Arms 2a or 2b (intervention arms) to invite these patients to participate in our study.

Subject Compensation
Subjects will not be financially compensated for their participation.

Procedures
Recruitment: The Orthopedic surgery scheduler will help the clinical research coordinator (CRC) identify appropriate patients who are planning inpatient joint replacement at UPHS. The RAPT score will be collected by the surgery scheduler and entered into EPIC. A copy of the RAPT tool has been attached this is submission. The RAPT tool is a validated measure that patients are asked to complete as part of routine care.

Randomization: Patients will be randomized 1:1 to usual care (Arm 1) and intervention (Arms 2a and 2b). Patients randomized to the intervention will be randomized 1:1 to Arm 2a remote monitoring or Arm 2b remote monitoring + goal-setting and social support. Both intervention arms will receive the intervention for 6 weeks post-surgery. Randomization will be stratified by hospital (PPMC vs PAH), and surgery type (hip vs. knee) . Patients will be randomized via the Way to Health platform prior to outreach. Patients will be randomized prior to outreach with a waiver of consent.

Enrollment: Patients that are randomized to the intervention Arms 2a and 2b will be invited to join our study by mailed invitation followed by phone calls and in person recruitment. These patients will be explained the benefits of going home after surgery and the ways in which we will provide (automated) support for the patient when they are home after surgery. Patients randomized to the intervention Arms 2a and 2b will still have to opt-in to the study. The enrolled patients in Arms 2a and 2b will receive a physical activity monitor, daily pain score tracking through bi-directional text messaging, milestone messaging, non-adherence messaging, and connection to clinicians as need. Patients enrolled in Arm 2b, remote monitoring + goal-setting and social support, will also receive nudge messaging to increase step count, feedback with motivational messages of patient progress, and social nudges from a support partner. The patients in Arms 2a and 2b will receive the automated feedback for 6 weeks post-surgery.

Intervention: The flow of the intervention is as follows: Patients Arm 2a and 2b can select to have the activity tracker mailed to their home for the patient to set-up on their own or to have the activity tracker set up in-person post-surgery at the hospital, depending on the patients comfort level with technology. The activity tracker will actively track daily step count after discharge. The activity tracker will send daily step count directly to the Way to Health platform. For 2 weeks post-surgery, patients will be asked to text-in their daily pain score, starting the day after discharge. Based on results from pilot testing daily pain scores from 14 patients, pain scores normalize after 2-3 weeks. The bi-directional text messages will be sent via the Way to Health platform and will say, "Good morning from HomeConnect+ On a scale of 1-10, how would you rate your pain today? (0=no pain, 10 = worst possible pain). Please respond with only the number." If a participant does not have a smartphone (with texting capabilities and access to the app for the activity tracker) we will provide one for them at the same time the patient receives their pedometer. Patients in Arm 2a and 2b will also receive milestone messaging for recovery developed by the Orthopedics service line 1 to 3 times a week. For example, "Hi from the HomeConnect+ team! Continue to manage pain, inflammation and swelling with pain medication, ice, and rest." For all enrolled patients, we will send an in-basket message to the clinical care team of patients enrolled in Arm 2a and 2b (intervention arms) to inform the care team that this patient has enrolled in HomeConnect+, a program to encourage the patient to go home after surgery. If a patient experiences a decrease in their average step count from last week AND an increase in their average pain score from last week, study staff will notify their clinical team via an EPIC in-basket message. Lastly participants will be contacted if they do not sync their activity tracker data for at least 3 days. Patients enrolled in Arm 2b, remote monitoring + goal-setting and social support will also receive the following: nudge messaging to increase step count, feedback with motivational messages of patient progress, and social nudges from a support partner. For patients in Arm 2b, the patients average step count for the second week will be considered the patients baseline. Patients will be rewarded levels based on their average increase in step count each week. The levels a

patient can be rewarded are: Bronze, Silver, Gold and Platinum. All patients will start at Bronze at baseline. If a patient increases their weekly average step count by 5% or more, the patient will move up a level. The patient will receive the following message, Hi (Participant Name) Congratulations! Your average step count this week increased from last week. You are now Silver status! If your average step count is (#) next week, you will move to Gold status! If the patient increases their weekly average step count by less than 5% they stay at the same level. The patient will receive the following message, Hi (Participant Name) Congratulations! Your average step count this week increased from last week. Keep up the good work and you can move to the next level next week! If a patients weekly average step count is less than the prior week, they move down a level. The patient will receive the following message, Hi (Participant Name) we've noticed that your average step count this week is less than last week, we will be calling you shortly to see if everything is okay. When enrolling in the program, the participants randomized to Arm 2b (remote monitoring + goal-setting and social support) will be asked to identify a social support. The social support will also receive the recovery feedback messages, such as " Your friend (Participant Name) achieved their weekly goal and have moved up a level. Send your congratulations and encourage them to keep up the good work! -HomeConnect." The social support will also receive an alert if the patient does not sync their activity tracker for at least 4 days.

Analysis: Chart review will be conducted for all patients (Arm 1, 2a and 2b) to determine patient discharge disposition and clinical outcomes post-surgery, such as TUG score. For the patients in the control arm (Arm 1), the data sources that will be collected include: Name, age, gender, surgery type, surgery location (hospital), RAPT score, discharge disposition, hospital readmissions, Physical Therapy measurements such as TUG score and Hoos/Koos. All of these sources are already collected as part of the patient routine standard care. All of these sources are also part of standard QI procedures conducted by the service line. The same data sources will be collected for the patients randomized to the intervention arms (Arm2a and 2b) as the control arm (Arm 1). If a patient is discharged to a SNF or inpatient rehab this will collected from the patients EMR (note above list of data sources and discharge disposition). Patients in Arm 2a and 2b who enrolled in Home Connect+ will receive a follow-up survey at the end of the program asking about their experience with HomeConnect.

Analysis Plan

1.1 Power and Sample Size

We expect the rate of discharge to home to be approximately 53% through usual care. With 300 patients we will have at least 80% power to detect an increase of 15% among those receiving the intervention, using a two-sided test with a significance level of 0.05. We anticipate approximately 35 patients in arms 2a and 2b. Based on a standard deviation in average daily step count of 1200 with a two sided alpha of 0.05, we have 80% power to detect an increase in average daily step count of 800 from week 2 to week 6 in arm 2b as compared to arm 2a.

1.2 Data analysis

We expect the rate of discharge to home to be approximately 53% through usual care. With 300 patients we will have at least 80% power to detect an increase of 15% among those receiving the intervention, using a two-sided test with a significance level of 0.05. We anticipate approximately 35 patients in arms 2a and 2b. Based on a standard deviation in average daily step count of 1200 with a two sided alpha of 0.05, we have 80% power to detect an increase in average daily step count of 800 from week 2 to week 6 in arm 2b as compared to arm 2a.

Data Confidentiality

Paper-based records will be kept in a secure location and only be accessible to personnel involved in the study. Computer-based files will only be made available to personnel involved in the study through the use of access privileges and passwords. Prior to access to any study-related information, personnel will be required to sign statements agreeing to protect the security and confidentiality of identifiable information. Wherever feasible, identifiers will be removed from study-related information.

Subject Confidentiality

The initial patient information collected for screening and recruitment will consist of name, address, phone number, demographic information such as race. As our patient population is patients in the UPHS health system, the majority of this information will come from Electronic Chart reviews. Database Security/Protection against Risk: To assure that patient, physician and other informant confidentiality is preserved, individual identifiers (such as name and medical record number) are stored in a single password protected system that is accessible only to study research, analysis and IT staff. This system is hosted on site at The University of Pennsylvania (UPenn) and is protected by a secure firewall. Once a participant is in this system, they will be given a unique study identification number (ID). Any datasets and computer files that leave the firewall will be stripped of all identifiers and individuals will be referred to by their study ID. The study ID will also be used on all analytical files. Please see attached document (WTH database security grant-protocol text FINAL (2)) for full database security details. RedCap will also be used for this study. REDCap is a secure web application for building and managing online surveys and databases. The institution installing REDCap will store all data captured in REDCap on its own servers. Therefore all project data is stored and hosted there at the local institution (University of Pennyslvania), and no project data is ever transmitted at any time by REDCap from that institution to another institution or organization. The pedometer monitoring devices will provide adherence data from each participant. This information is transmitted via from the pedometer to a smartphone app. Nokias privacy measures can be found here: http://www.nokia.com/en_int/privacy Patients in the intervention arm that are provided a smart phone for the study will be allowed to keep the smart phone when the study is over. Research material that is obtained will be used for research purposes only. The same procedure used for the analysis of automated data sources to ensure protection of patient information will be used for pedometer data, in that patient identifiers will be used only for linkage purposes or to contact patients. The study identification number, and no other identifying information, will be used on all data collection instruments. All study staff will be reminded to appreciate the confidential nature of the data collected and contained in these databases.

Database Security/Protection Against Risk

The UPENN Biomedical Informatics Consortium (BMIC) will be the hub for the hardware and database infrastructure that will support the project and where the Way to Health web portal is based. The BMIC is a joint effort of the University of Pennsylvania's Abramson Cancer Center, the Cardiovascular Institute, the Department of Pathology, and the Leonard Davis Institute. The BMIC provides a secure computing environment for a large volume of highly sensitive data, including clinical, genetic, socioeconomic, and financial information. Among the IT projects currently managed by BMIC are: (1) the capture and organization of complex, longitudinal clinical data via web and clinical applications portals from cancer patients enrolled in clinical trials; (2) the integration of genetic array databases and clinical data obtained from patients with cardiovascular disease; (3) computational biology and cytometry database management and analyses; (4) economic and health policy research using Medicare claims from over 40 million Medicare beneficiaries. BMIC requires all users of data or applications on BMIC servers to complete a BMIC-hosted cybersecurity awareness course annually, which stresses federal data security policies under data use agreements with the university. Curriculum includes HIPAA training and covers secure data transfer, passwords, computer security habits and knowledge of

what constitutes misuse or inappropriate use of the server. We will implement multiple, redundant protective measures to guarantee the privacy and security of the participant data. All investigators and research staff with direct access to the identifiable data will be required to undergo annual responsible conduct of research, cybersecurity, and Health Insurance Portability and Accountability Act certification in accordance with University of Pennsylvania regulations. All data for this project will be stored on the secure/firewalled servers of the BMIC Data Center, in data files that will be protected by multiple password layers. These data servers are maintained in a guarded facility behind several locked doors, with very limited physical access rights. They are also cyber-protected by extensive firewalls and multiple layers of communication encryption. Electronic access rights are carefully controlled by University of Pennsylvania system managers. We believe this multi-layer system of data security, identical to the system protecting the University of Pennsylvania Health Systems medical records, greatly minimizes the risk of loss of privacy. Each subject will be assigned a unique identifier without identifying information, and data will be entered into an electronic database using only the unique identifier. Only trained study staff will have access to the code that links the unique identifier to the subjects identity. Electronic data will be stored on secure, password-protected firewalled servers at UPENN.

Sensitive Research Information

This Research does not involve collection of sensitive information about the subjects that should be excluded from the electronic medical record.

Subject Privacy

We will only interact with the subsample of subjects with which we plan to enroll in the study. With these subjects, we will conduct phone calls in a private area. When we call subjects, we will confirm the identify before beginning the recruitment process. When conducting the in-person 4-month visit, we will conduct blood pressure measurements and surveys in a private office location, with only one subject at a time.

Data Disclosure

The following entities, besides the members of the research team, may receive PHI for this research study: Nokia the company which records the responses from the pedometer. Daily activity data will be stored on the participants personal cellphone in the Nokia App. Nokias privacy measures can be found here: http://www.nokia.com/en_int/privacy Other entities include The Office of Human Research Protections at the University of Pennsylvania Federal and state agencies (for example, the Department of Health and Human Services, the National Institutes of Health, and /or the Office for Human Research Protections), or other domestic or foreign government bodies if required by law and/ or necessary for oversight purposes.

Protected Health Information/Data Protection

- Name
- Street address, city, county, precinct, zip code, and equivalent geocodes
- Telephone and fax numbers
- Electronic mail addresses
- Medical record numbers

Consent Process

1.1 Overview

This study is minimal risk as all patients are receiving standard clinical care. This study was designed in collaboration with the Penn Medicine Musculoskeletal Service Line. The intervention is clinically appropriate and aligns with the standard patient clinical care, and the consent process itself may influence the outcome of our study. The purpose of the HomeConnect+ program is to encourage patients to go home after surgery. The invitation to be part of HomeConnect+ includes language to nudge the patient to choose home as their discharge goal. Explaining the program to a potential participant randomized to the Usual Care arm may influence the patients decision making process for discharge disposition. It would be impractical to obtain a written signature for consent and HIPPA authorization. We are requesting a waiver of the requirement to document consent and HIPPA authorization with signature for participants enrolled in the intervention since we believe the research presents no more than minimal risk of harm to subjects and involves no procedures for which written consent is normally required outside of the research context. Participation in the intervention is completely voluntary. Participants randomized to the intervention arms still need to opt-in to the study. All participants receive standard clinical care. Participants randomized to the intervention arms will be informed that this program is voluntary and that they can stop participating in the program (stop using the activity pedometer) at any time.

1.2 Children and Adolescents

Not applicable

1.3 Adult Subjects Not Competent to Give Consent

Waiver of consent is being requested.

Waiver of Consent

1.1 Minimal Risk

This study is minimal risk as all patients are receiving standard clinical care. This study was designed in collaboration with the Penn Medicine Musculoskeletal Service Line. The intervention is clinically appropriate and aligns with the standard patient clinical care.

1.2 Impact on Subjects Rights and Welfare

Participation in the intervention is completely voluntary. Participants randomized to the intervention arms still need to opt-in to the study. All participants receive standard clinical care.

1.3 Waiver Essential to Research

The consent process itself may influence the outcome of our study. The purpose of the HomeConnect+ program is to encourage patients to go home after surgery. The invitation to be part of HomeConnect+ includes language to nudge the patient to choose home as their discharge goal. Explaining the program to a potential participant randomized to the Usual Care arm may influence the patients decision making process for discharge disposition. It would be impractical to obtain a written signature for consent and HIPPA authorization. We are requesting a waiver of the requirement to document consent and HIPPA authorization with signature for participants enrolled in the intervention since we believe the research presents no more than minimal risk of harm to subjects and involves no procedures for which written consent is normally required outside of the research context.

1.4 Written Statement of Research
None

Potential Study Risks
As this study does not involve changes to standard patient care or medical decision making and only tests the use of remote monitoring approaches to encouraging patients to go home after surgery, we consider this study minimal risk. The primary risk would be from a breach of confidentiality involving medical records reviews and monitoring of activity tracker, or text messaging which will be maintained on the Way to Health platform. This risk has been mitigated by extensive privacy protection protocols, a highly secure data storage system, and a plan to remove identifiers from the data wherever possible. In addition, all personnel will be held to high standards of upholding confidentiality and safeguarding patient privacy.

Potential Study Benefits
The immediate benefits of this study for participants may include an improvement in recovery, both due to going home after surgery which has been proven effective in improving patient outcomes and due to feedback and milestone messaging. It is possible that the benefits for many participants will be minimal. However, as mentioned, we believe the risks are also minimal. Overall the risk to benefit ratio is favorable given the long term potential of improvement for health and health related behaviors Participants in this study may not receive any direct benefits. Some may benefit directly by going home after surgery, receiving extra support when recovering at home from the remote monitoring through the feedback and milestone messaging or feel more connected to their clinical care team. The control group is unlikely to directly benefit, as this group will continue to simply receive usual care. Knowledge gained from the study will assist in development of interventions in other post-acute care populations. The potential public health impact of a successful intervention to improve recovery for LEJR patients is enormous and could reduce the number of readmissions and related costs in the United States each year. The risks of loss of confidentiality are minimal in this study. Thus, the benefits of this research to the participants studied, and to society at large, far surpass the risks.

Data and Safety Monitoring
The study is minimal risk to participants and therefore the Principal Investigator will monitor the safety of this study.

Risk/Benefit Assessment
Hip and knee replacements are the most common inpatient surgery for Medicare beneficiaries, with substantial cost during the hospitalization and through post-acute care, where there is substantial variability. This study is designed to test an intervention that incorporates many components that we have previously tested through rapid-cycle experimentation that we believe can improve the value and outcomes for patients who received joint replacement surgery. We believe that the combination of these approaches in this study will prove the research and public health communities with important information that can lead to broad generalizability in post-acute after for LEJR patients, as these types of interventions could be set up by healthcare system to be broadly utilized.

Final Protocol

****New changes from initial protocol notated in bold, parts removed from initial protocol notated in strikethrough**

Abstract
The goal of this study is to test the approach of automated hovering to encourage patients to be discharged to home safely after lower extremity joint replacement surgery. Among UPHS patients, we will target those undergoing hip or knee replacement surgery at PPMC and PAH. Our aim is to increase the number of patients that are discharged to home, by providing patients with a physical activity pedometer, daily pain score tracking through bi-directional text messaging, milestone and nudge messaging for recovery, social influence, and connection to clinicians as needed for 6 weeks after surgery. Eligible patients are age 18-85, with a Risk Assessment and Prediction Tool (RAPT) score of 6-8 and are scheduled to undergo lower-extremity joint replacement surgery. Patients will be randomized 1:1 to usual care (Arm 1) and intervention (Arms 2a and 2b). After enrollment with the monitoring device, patients in the intervention group will be randomized 1:1 to Arm 2a remote monitoring or Arm 2b remote monitoring + goal-setting and social support). Both intervention arms will receive the intervention for 6 weeks post-surgery.

Study Instruments
All subjects enrolled in either Arm 2a or 2b will receive a follow-up survey at the end of the program asking about their experience with HomeConnect+.

Group Modifications
Only participants enrolled in one of the intervention arms (2a or 2b) will receive an activity monitor, daily monitoring, milestone messaging and connection to clinicians as needed for 6 weeks after their surgery. Participants enrolled in Arm 2b will additionally receive nudge messaging to increase step count, feedback with motivational messages of patient progress, and social nudges from a support partner.

Administration of Surveys
All subjects enrolled in either Arm 2a or 2b will receive a follow-up survey at the end of the program asking about their experience with HomeConnect+. This survey will be accessible via a text link and will take approximately 5 minutes to complete.

Objectives
1.4 Objectives
The specific aims of this study are to:
Aim 1: ~~provide~~ **Evaluate the effectiveness of offering automated hovering with activity monitoring and motivational messages (arm 2) to encourage patients to be discharged to home after hip or knee replacement surgery compared to usual care (arm 1).**~~remote monitoring with nudges to patients, support partner, and staff to encourage patient discharge to home.~~
Aim 2: **Among those receiving automated hovering, evaluate whether providing goal setting with social influence (arm 2a) increase activity levels compared to feedback alone (arm 2b).** ~~Obtain additional data about the implications of activity monitoring data and clinical outcomes.~~

1.5 Primary Outcome Variable
The primary outcome will be **the discharge status (home vs not home, e.g. skilled nursing facility or in-patient rehabilitation facility).** ~~percent of patients that are discharged to home.~~ An additional primary outcome for intervention patients is the **change in average daily steps from baseline (week 2) to the end of the intervention (week 6).** ~~percent step increase.~~

1.6 Secondary Outcome Variable(s)
The secondary outcomes will **number of** be days at home **and Timed Up and Go (**TUG**)** score **measured after surgery.**

Additional exploratory outcomes will include patient-reported outcomes **(HOOS/KOOS scores)**, patient ~~experience~~ **satisfaction with the intervention (measured by post-intervention text message), 45-day-all-cause** readmissions, overall **health care** utilization **(hospitalizations, emergency department visits, outpatient visits, physical therapy)**, length of **hospital** stay, and time to home **from day of surgery**~~.,~~ ~~increase in average daily step count from week 2 to week.~~

Background
1.1 Program Goals
Hip and knee replacements are the most common inpatient surgery for Medicare beneficiaries, with substantial cost during the hospitalization and through post-acute care, where there is substantial variability. The University of Pennsylvania Health System (UPHS) participates in voluntary Medicare, IBC, and Horizon bundled payment programs, in addition to being subject to readmission penalties by Medicare. UPHS discharges about 70% of patients to home (53% of patients with RAPT score 6-8), and patients may not engage in the recommended physical therapy activities to achieve optimal clinical outcomes. For this patient population, alternatives to discharge to home are inpatient rehab facility and a Skilled Nursing Facility or SNF. Patients that are discharged to inpatient rehab or SNF are more likely to have post-discharge severe adverse events and unplanned readmissions than patients that are discharged to home post-surgery.

During the last year, our team conducted contextual inquiry and rapid cycle experimentation with the goal of improving the value and outcomes for patients who received joint replacement surgery. This work was conducted as part of a QI initiative with the Penn Medicine MSK service line. We first conducted chart review of discharge disposition by RAPT score for LEJR patients in 2016. We correlated the RAPT score with patient intention and their ultimate discharge destination. Then, we conducted observations and interviews of patients and Orthopedics Care Team members about discharge disposition decision process. The goal was to better understand the existing processes to identify areas for improvement. From this work we learned that patient preference was important to disposition and home was not necessarily offered as the preferred option before surgery.

From shadowing patients pre-op appointments with Social Work as they discussed disposition goals with LEJR patients we discovered that Social Work can influence pre-op goal setting. We also discovered that offering home as a default choice (instead of an active choice) for patients with RAPT score 9 (low risk) will increase the number of patients discharged to home (from 68% to 73%). In addition, we learned that by giving physical therapists information about step counts and daily pain scores, did not increase their confidence in discharging patients from home PT sooner. As part of the rapid cycle experimentation we provided remote monitoring and feedback via text messaging in a program called HomeConnect Pilot to patients undergoing LEJR surgery with RAPT score 6-8 from March to June 2017. The HomeConnect Pilot, offered by the Orthopedics Department at Penn Medicine, was designed to help keep the patient connected with their care team after surgery through the use of a remote monitoring devices (pedometer) and text messaging, such as daily pain score. The

HomeConnect program lasted for 30 days after surgery and was offered as a voluntary program in addition to the patient's usual care. We obtained verbal consent from patients for text messaging as guided by the Privacy Officer of Penn Medicine, as has been done for other text messaging interventions. In this pilot, 14 medium-risk patients (RAPT 6-8) were enrolled in Way to Health before surgery and showed the feasibility and acceptability of automated hovering with 73% using the devices with robust data about daily activity and pain scores. 79% of patients enrolled in the platform went home, which was higher than the baseline rate of 53% for medium-risk patients (RAPT 6-8). Through this pilot we also discovered that the LEJR patient population can safely use the pedometer. We also discovered that collecting daily step count and daily text messaging with this population is feasible. Monitoring of daily step count and pain score for the patients enrolled in the HomeConnect Pilot was done through the Way to Health platform. The Way to Health platform is a PennMedicine platform, hosted on site at the University of Pennsylvania and is protected by a secure firewall. All patient information was stored in EPIC, RedCap, or Way to Health, all HIPAA compliant platforms. For the purposes of dissemination, no patient identifiers will be used and data will be provided in the aggregate.

Statistical Considerations

1.1 Power and sample size

We expect the rate of discharge to home to be approximately 53% ~~through~~ **for patients receiving** usual care. With 300 patients we will have at least 80% power to detect an increase of ~~15~~ **16**% among those receiving the intervention, using a two-sided test with a significance level of 0.05.

Among the 150 patients who are offered the intervention, w~~We~~ **e anticipate approximately** 70 patients will enroll in and receive hovering. They will be randomized in a 1:1 ratio to **35 patients** each **in arms 2a and 2b. Based on a standard deviation in average daily step count of 1200 with a two sided alpha of 0.05, we have 80% power to detect an increase in average daily step count of 800 from week 2 to week 6 in arm 2b as compared to arm 2a**.

1.2 Data analysis

We expect the rate of discharge to home to be approximately 53% ~~through~~ **for patients receiving** usual care. With 300 patients we will have at least 80% power to detect an increase of ~~15~~ **16**% among those receiving the intervention, using a two-sided test with a significance level of 0.05. **We will compare discharge status proportion using the chi-squared test of proportions.**

Among the 150 patients who are offered the intervention, w~~W~~e anticipate approximately **70 patients will enroll in and receive hovering. They will be randomized in a 1:1 ratio to** 35 patients **each** in arms 2a and 2b. Based on a standard deviation in average daily step count of 1200 with a two sided alpha of 0.05, we have 80% power to detect an increase in average daily step count of 800 from week 2 to week 6 in arm 2b as compared to arm 2a. **We will use the independent group t-test to compare the difference in average daily step count from baseline to the end of the intervention between arms 2a and 2b.**

Study Design

1.1 Design

In this study, the Orthopedic surgery scheduler will help the clinical research coordinator (CRC) identify appropriate patients who are planning inpatient joint replacement at UPHS. Patients will be randomized 1:1 to usual care (Arm 1) and intervention (Arms 2a and 2b). Patients randomized to the intervention will be randomized 1:1 to Arm 2a remote monitoring or Arm 2b remote monitoring + goalsetting and social support. Both intervention arms will receive the intervention for 6 weeks post-surgery.

Randomization will be stratified by hospital (PPMC vs PAH), and surgery type (hip vs. knee). Patients will be randomized via the Way to Health platform prior to outreach. Patients will be randomized prior to outreach with a waiver of consent for the following reasons: 1) the study is low risk 2) the patients randomized to Arm 1 (usual care) receive standard and routine clinical care, and this study does not violate their welfare, 3) the consent process itself may affect the outcome of our study.

To further explain number 3, patients that are randomized to Arms 2a and 2b (automated hovering) will be invited to join our study by mail and phone. These patients will be explained the benefits of going home after surgery and the ways in which we will provide (automated) support for the patient when they are home after surgery. Since the explanation of our study encourages patients to go home after surgery (our primary outcome), and we are not interacting with all patients, a waiver of consent is needed. Patients randomized to Arms 2a and 2b (intervention arms) will still have to opt-in to the intervention. The enrolled patients in Arms 2a and 2b will receive a physical activity monitor, daily pain score tracking through bi-directional text messaging, milestone messaging, non-adherence messaging, and connection to clinicians as need. Patients enrolled in Arm 2b, remote monitoring + goal-setting and social support, will also receive nudge messaging to increase step count, feedback with motivational messages of patient progress, and social nudges from a support partner. The patients in Arms 2a and 2b will receive the automated feedback for 6 weeks post-surgery. A detailed outline of study design can be found in the procedures sections.

<u>1.2 Consent Process</u>

This study is minimal risk as all patients are receiving standard clinical care. This study was designed in collaboration with the Penn Medicine Musculoskeletal Service Line. The intervention is clinically appropriate and aligns with the standard patient clinical care, and the consent process itself may influence the outcome of our study. The purpose of the HomeConnect+ program is to encourage patients to go home after surgery. The invitation to be part of HomeConnect+ includes language to nudge the patient to choose home as their discharge goal. Explaining the program to a potential participant randomized to the Usual Care arm may influence the patients decision making process for discharge disposition. It would be impractical to obtain a written signature for consent and HIPPA authorization. We are requesting a waiver of the requirement to document consent and HIPPA authorization with signature for participants enrolled in the intervention since we believe the research presents no more than minimal risk of harm to subjects and involves no procedures for which written consent is normally required outside of the research context. Participation in the intervention is completely voluntary. Participants randomized to the intervention arms still need to opt-in to the study. All participants receive standard clinical care. Participants randomized to the intervention arms will be informed that this program is voluntary and that they can stop participating in the program (stop using the activity pedometer) at any time.

Study Duration

The duration of participation for the intervention will be 6 weeks after discharge from the hospital. We expect the total duration of the study to last 8 months for all patients to complete the intervention, however time from schedule to surgery is highly variable for LEJR. We will recruit patients from December 2017 through June 2018. We estimate that the study will be completed by September 2018.

Resources Necessary for Human Research Protections

The project will take place at the Leonard Davis Institute Center for Health Incentives and Behavioral Economics (LDI CHIBE) at the University of Pennsylvania (UPENN). The team includes investigators experienced in clinical medicine, health behavior interventions, clinical trials, behavioral economics, cost-effectiveness analysis, and program evaluation. Our partnership combines the resources and capabilities of a major university (the Wharton School and the Perelman School of Medicine at the

University of Pennsylvania). This study will have the support of multiple PIs: Dr. Shivan Mehta, MD, MBA, MSHP, is the Director of Operations for the Penn Medicine Center for Health Care Innovation. Dr. Kevin Volpp, MD, PhD, directs the LDI CHIBE and the NIA-funded PENN-CMU Roybal P30 Center on Behavioral Economics and Health and is a Professor of Medicine at the Perelman School of Medicine (SOM) and Professor of Health Care Management at the Wharton School at UPENN. David Asch, MD, MBA - is Executive Director of the Penn Medicine Center for Innovation, Professor of Health Care Management and Economics and Professor of Operations and Information Management at Wharton and Professor of Medicine at Perelman. This study will be supported on a secure web portal on the Way to Health platform, modified to the specifications of this study.

Target Population
Eligibility Criteria: UPHS patients between the ages of 18-85 with a Risk Assessment and Prediction Tool (RAPT) of 6-8 and scheduled to undergo lower-extremity joint replacement surgery. The total target enrollment will be 300 patients. 150 in usual care (Arm 1) and 150 in the intervention arms (Arm 2a and 2b). We estimate that roughly 70 participants in the intervention arms (35 per arm) will be active in this study. This number is based on the estimation that 60% of the patients randomized to the intervention will opt-in to the study and roughly 17% of patients in this population do not proceed with surgery.

Subjects Enrolled by Penn Researchers
300

Subjects Enrolled by Collaborating Researchers
0

Accrual
Participants in this study will be undergoing LEJR surgery at Penn Medicine. They will be identified by the Orthopedic surgery schedulers and shared with our study staff. A study coordinator will review the list of patients to determine eligibility. If a patient meets the minimal requirements they will be entered into the Way to Health platform for randomization. The study coordinator will send a mailed invitation and brochure followed by reaching out via phone to the patients randomized to Arm 2a (remote monitoring) or Arm ~~3~~**2b** (remote monitoring + goal -setting and social support) to invite these patients to partake in our study.

Key Inclusion Criteria
Eligibility Criteria: UPHS patients between the ages of 18-85 with a Risk Assessment and Prediction Tool (RAPT) of 6-8 and scheduled to undergo primary lower-extremity joint replacement surgery. Patients that enroll in the HomeConnect program and are discharge to a SNF can still be part of the HomeConnect program; we will still track their daily step count and pain score. Patients with a RAPT score of 6-8 are considered low risk and able to go home after surgery, however there are things that may occur in the patients care post-surgery that may change their discharge disposition to SNF or an inpatient rehab facility. For this reason, we have included days at home and length of stay as part of our secondary outcomes.

Key Exclusion Criteria
Exclusion Criteria: Patients will be excluded if they do not meet all of the inclusion criteria, or if they have bilateral or revision surgery, dementia, end stage renal disease, cirrhosis, metastatic cancer, ~~non-English speaker~~, or other physical impairment (ex. amputation).

Vulnerable Populations
No vulnerable Populations are included in the research study.

Populations Vulnerable to Undue Influence or Coercion
We are not specifically targeting any vulnerable populations.

Subject Recruitment
The Orthopedic surgery scheduler will help the clinical research coordinator (CRC) identify appropriate patients who are planning inpatient joint replacement at UPHS. Patients will be randomized 1:1 to usual care (Arm 1) and intervention (Arm 2a remote monitoring and Arm 2b remote monitoring + goalsetting and social support). If a patient meets the minimal requirements they will be entered into the Way to Health platform for randomization prior to outreach. The study coordinator will send a mailed invitation and then reach out via phone or in-person recruitment (up to 4 phone calls) to the patients randomized to Arms 2a or 2b (intervention arms) to invite these patients to participate in our study.

Subject Compensation
Subjects will not be financially compensated for their participation.

Procedures
Recruitment: The Orthopedic surgery scheduler will help the clinical research coordinator (CRC) identify appropriate patients who are planning inpatient joint replacement at UPHS. The RAPT score will be collected by the surgery scheduler and entered into EPIC. A copy of the RAPT tool has been attached this is submission. The RAPT tool is a validated measure that patients are asked to complete as part of routine care.

Randomization: Patients will be randomized 1:1 to usual care (Arm 1) and intervention (Arms 2a and 2b). Patients randomized to the intervention will be randomized 1:1 to Arm 2a remote monitoring or Arm 2b remote monitoring + goal-setting and social support. Both intervention arms will receive the intervention for 6 weeks post-surgery. Randomization will be stratified by hospital (PPMC vs PAH), and surgery type (hip vs. knee) . Patients will be randomized via the Way to Health platform prior to outreach. Patients will be randomized prior to outreach with a waiver of consent.

Enrollment: Patients that are randomized to the intervention Arms 2a and 2b will be invited to join our study by mailed invitation followed by phone calls and in person recruitment. These patients will be explained the benefits of going home after surgery and the ways in which we will provide (automated) support for the patient when they are home after surgery. Patients randomized to the intervention Arms 2a and 2b will still have to opt-in to the study. The enrolled patients in Arms 2a and 2b will receive a physical activity monitor, daily pain score tracking through bi-directional text messaging, milestone messaging, non-adherence messaging, and connection to clinicians as need. Patients enrolled in Arm 2b, remote monitoring + goal-setting and social support, will also receive nudge messaging to increase step count, feedback with motivational messages of patient progress, and social nudges from a support partner. The patients in Arms 2a and 2b will receive the automated feedback for 6 weeks post-surgery.

Intervention: The flow of the intervention is as follows: Patients Arm 2a and 2b can select to have the activity tracker mailed to their home for the patient to set-up on their own or to have the activity tracker set up in-person post-surgery at the hospital, depending on the patients comfort level with technology. **For the patients that do not have access to a smartphone, a smartphone will be provided for the study.** The activity tracker will actively track daily step count after discharge. The activity tracker will send daily step count directly to the Way to Health platform. For 2 weeks post-surgery, patients will be asked to text-in their daily pain score, starting the day after discharge. Based on results from pilot testing daily pain scores from 14 patients, pain scores normalize after 2-3 weeks. The bi-directional text messages will be

sent via the Way to Health platform and will say, "Good morning from HomeConnect+ On a scale of 1-10, how would you rate your pain today? (0=no pain, 10 = worst possible pain). Please respond with only the number." If a participant does not have a smartphone (with texting capabilities and access to the app for the activity tracker) we will provide one for them at the same time the patient receives their pedometer. Patients in Arm 2a and 2b will also receive milestone messaging for recovery developed by the Orthopedics service line 1 to 3 times a week. For example, "Hi from the HomeConnect+ team! Continue to manage pain, inflammation and swelling with pain medication, ice, and rest." For all enrolled patients, we will send an in-basket message to the clinical care team of patients enrolled in Arm 2a and 2b (intervention arms) to inform the care team that this patient has enrolled in HomeConnect+, a program to encourage the patient to go home after surgery. If a patient experiences a decrease in their average step count from last week AND an increase in their average pain score from last week, study staff will notify their clinical team via an EPIC in-basket message. Lastly participants will be contacted if they do not sync their activity tracker data for at least 3 days. Patients enrolled in Arm 2b, remote monitoring + goal-setting and social support will also receive the following: nudge messaging to increase step count, feedback with motivational messages of patient progress, and social nudges from a support partner. For patients in Arm 2b, the patients average step count for the second week will be considered the patients baseline. Patients will be rewarded levels based on their average increase in step count each week. The levels a patient can be rewarded are: Bronze, Silver, Gold and Platinum. All patients will start at Bronze at baseline. If a patient increases their weekly average step count by 5% or more, the patient will move up a level. The patient will receive the following message, Hi (Participant Name) Congratulations! Your average step count this week increased from last week. You are now Silver status! If your average step count is (#) next week, you will move to Gold status! If the patient increases their weekly average step count by less than 5% they stay at the same level. The patient will receive the following message, Hi (Participant Name) Congratulations! Your average step count this week increased from last week. Keep up the good work and you can move to the next level next week! If a patient’s weekly average step count is less than the prior week~~, they move down a level. T~~**t**he patient will receive the following message, Hi (Participant Name) we’ve noticed that your average step count this week is less than last week, we will be calling you shortly to see if everything is okay. When enrolling in the program, the participants randomized to Arm 2b (remote monitoring + goal-setting and social support) will be asked to identify a social support. The social support will also receive the recovery feedback messages, such as " Your friend (Participant Name) achieved their weekly goal and have moved up a level. Send your congratulations and encourage them to keep up the good work! -HomeConnect." The social support **partner for the patients in arm 2b** will also receive an alert if the patient does not sync their activity tracker for at least 4 days. **Patients in the intervention arms (2a and 2b) will receive a notification if they do not sync their activity tracker for 3 days, and if they do not text their daily pain score for 3 days. If a participant has not synced the activity tracker for over a week, a letter will be mailed with detailed syncing instructions.**

Analysis: Chart review will be conducted for all patients (Arm 1, 2a and 2b) to determine patient discharge disposition and clinical outcomes post-surgery, such as TUG score. For the patients in the control arm (Arm 1), the data sources that will be collected include: Name, age, gender, surgery type, surgery location (hospital), RAPT score, discharge disposition, hospital readmissions, Physical Therapy measurements such as TUG score and Hoos/Koos. All of these sources are already collected as part of the patient routine standard care. All of these sources are also part of standard QI procedures conducted by the service line. The same data sources will be collected for the patients randomized to the intervention arms (Arm2a and 2b) as the control arm (Arm 1). If a patient is discharged to a SNF or inpatient rehab this will collected from the patients EMR (note above list of data sources and discharge disposition). Patients in Arm 2a and 2b who enrolled in Home Connect+ will receive a follow-up survey at the end of the program asking about their experience with HomeConnect.

Analysis Plan

1.1 Power and sample size

We expect the rate of discharge to home to be approximately 53% ~~through~~ **for patients receiving** usual care. With 300 patients we will have at least 80% power to detect an increase of ~~15~~ **16**% among those receiving the intervention, using a two-sided test with a significance level of 0.05.

Among the 150 patients who are offered the intervention, w~~W~~**e anticipate approximately** 70 patients will enroll in and receive hovering. They will be randomized in a 1:1 ratio to **35 patients** each **in arms 2a and 2b. Based on a standard deviation in average daily step count of 1200 with a two sided alpha of 0.05, we have 80% power to detect an increase in average daily step count of 800 from week 2 to week 6 in arm 2b as compared to arm 2a**.

1.2 Data analysis

We expect the rate of discharge to home to be approximately 53% ~~through~~ **for patients receiving** usual care. With 300 patients we will have at least 80% power to detect an increase of ~~15~~ **16**% among those receiving the intervention, using a two-sided test with a significance level of 0.05. **We will compare discharge status proportion using the chi-squared test of proportions.**

Among the 150 patients who are offered the intervention, w~~W~~e anticipate approximately **70 patients will enroll in and receive hovering. They will be randomized in a 1:1 ratio to** 35 patients **each** in arms 2a and 2b. Based on a standard deviation in average daily step count of 1200 with a two sided alpha of 0.05, we have 80% power to detect an increase in average daily step count of 800 from week 2 to week 6 in arm 2b as compared to arm 2a. **We will use the independent group t-test to compare the difference in average daily step count from baseline to the end of the intervention between arms 2a and 2b.**

Data Confidentiality

Paper-based records will be kept in a secure location and only be accessible to personnel involved in the study. Computer-based files will only be made available to personnel involved in the study through the use of access privileges and passwords. Prior to access to any study-related information, personnel will be required to sign statements agreeing to protect the security and confidentiality of identifiable information. Wherever feasible, identifiers will be removed from study-related information.

Subject Confidentiality

The initial patient information collected for screening and recruitment will consist of name, address, phone number, demographic information such as race. As our patient population is patients in the UPHS health system, the majority of this information will come from Electronic Chart reviews. Database Security/Protection against Risk: To assure that patient, physician and other informant confidentiality is preserved, individual identifiers (such as name and medical record number) are stored in a single password protected system that is accessible only to study research, analysis and IT staff. This system is hosted on site at The University of Pennsylvania (UPenn) and is protected by a secure firewall. Once a participant is in this system, they will be given a unique study identification number (ID). Any datasets and computer files that leave the firewall will be stripped of all identifiers and individuals will be referred to by their study ID. The study ID will also be used on all analytical files. Please see attached document (WTH database security grant-protocol text FINAL (2)) for full database security details. RedCap will also be used for this study. REDCap is a secure web application for building and managing online surveys and databases. The institution installing REDCap will store all data captured in REDCap on its own servers. Therefore all project data is stored and hosted there at the local institution (University of Pennyslvania), and no project data is ever transmitted at any time by

REDCap from that institution to another institution or organization. The pedometer monitoring devices will provide adherence data from each participant. This information is transmitted via from the pedometer to a smartphone app. Nokias privacy measures can be found here: http://www.nokia.com/en_int/privacy Patients in the intervention arm that are provided a smart phone for the study will be allowed to keep the smart phone when the study is over. Research material that is obtained will be used for research purposes only. The same procedure used for the analysis of automated data sources to ensure protection of patient information will be used for pedometer data, in that patient identifiers will be used only for linkage purposes or to contact patients. The study identification number, and no other identifying information, will be used on all data collection instruments. All study staff will be reminded to appreciate the confidential nature of the data collected and contained in these databases.

Database Security/Protection Against Risk

The UPENN Biomedical Informatics Consortium (BMIC) will be the hub for the hardware and database infrastructure that will support the project and where the Way to Health web portal is based. The BMIC is a joint effort of the University of Pennsylvania's Abramson Cancer Center, the Cardiovascular Institute, the Department of Pathology, and the Leonard Davis Institute. The BMIC provides a secure computing environment for a large volume of highly sensitive data, including clinical, genetic, socioeconomic, and financial information. Among the IT projects currently managed by BMIC are: (1) the capture and organization of complex, longitudinal clinical data via web and clinical applications portals from cancer patients enrolled in clinical trials; (2) the integration of genetic array databases and clinical data obtained from patients with cardiovascular disease; (3) computational biology and cytometry database management and analyses; (4) economic and health policy research using Medicare claims from over 40 million Medicare beneficiaries. BMIC requires all users of data or applications on BMIC servers to complete a BMIC-hosted cybersecurity awareness course annually, which stresses federal data security policies under data use agreements with the university. Curriculum includes HIPAA training and covers secure data transfer, passwords, computer security habits and knowledge of what constitutes misuse or inappropriate use of the server. We will implement multiple, redundant protective measures to guarantee the privacy and security of the participant data. All investigators and research staff with direct access to the identifiable data will be required to undergo annual responsible conduct of research, cybersecurity, and Health Insurance Portability and Accountability Act certification in accordance with University of Pennsylvania regulations. All data for this project will be stored on the secure/firewalled servers of the BMIC Data Center, in data files that will be protected by multiple password layers. These data servers are maintained in a guarded facility behind several locked doors, with very limited physical access rights. They are also cyber-protected by extensive firewalls and multiple layers of communication encryption. Electronic access rights are carefully controlled by University of Pennsylvania system managers. We believe this multi-layer system of data security, identical to the system protecting the University of Pennsylvania Health Systems medical records, greatly minimizes the risk of loss of privacy. Each subject will be assigned a unique identifier without identifying information, and data will be entered into an electronic database using only the unique identifier. Only trained study staff will have access to the code that links the unique identifier to the subjects identity. Electronic data will be stored on secure, password-protected firewalled servers at UPENN.

Sensitive Research Information

This Research does not involve collection of sensitive information about the subjects that should be excluded from the electronic medical record.

Subject Privacy

We will only interact with the subsample of subjects with which we plan to enroll in the study. With these subjects, we will conduct phone calls in a private area. When we call subjects, we will confirm the identify before beginning the recruitment process. When conducting the in-person 4-month visit, we will conduct blood pressure measurements and surveys in a private office location, with only one subject at a time.

Data Disclosure

The following entities, besides the members of the research team, may receive PHI for this research study: Nokia the company which records the responses from the pedometer. Daily activity data will be stored on the participants personal cellphone in the Nokia App. Nokias privacy measures can be found here: http://www.nokia.com/en_int/privacy Other entities include The Office of Human Research Protections at the University of Pennsylvania Federal and state agencies (for example, the Department of Health and Human Services, the National Institutes of Health, and /or the Office for Human Research Protections), or other domestic or foreign government bodies if required by law and/ or necessary for oversight purposes.

Protected Health Information/Data Protection

- Name
- Street address, city, county, precinct, zip code, and equivalent geocodes
- Telephone and fax numbers
- Electronic mail addresses
- Medical record numbers

Consent Process

1.1 Overview

This study is minimal risk as all patients are receiving standard clinical care. This study was designed in collaboration with the Penn Medicine Musculoskeletal Service Line. The intervention is clinically appropriate and aligns with the standard patient clinical care, and the consent process itself may influence the outcome of our study. The purpose of the HomeConnect+ program is to encourage patients to go home after surgery. The invitation to be part of HomeConnect+ includes language to nudge the patient to choose home as their discharge goal. Explaining the program to a potential participant randomized to the Usual Care arm may influence the patients decision making process for discharge disposition. It would be impractical to obtain a written signature for consent and HIPAA authorization. We are requesting a waiver of the requirement to document consent and HIPAA authorization with signature for participants enrolled in the intervention since we believe the research presents no more than minimal risk of harm to subjects and involves no procedures for which written consent is normally required outside of the research context. Participation in the intervention is completely voluntary. Participants randomized to the intervention arms still need to opt-in to the study. All participants receive standard clinical care. Participants randomized to the intervention arms will be informed that this program is voluntary and that they can stop participating in the program (stop using the activity pedometer) at any time.

1.2 Children and Adolescents

Not applicable

1.3 Adult Subjects Not Competent to Give Consent
Waiver of consent is being requested.

Waiver of Consent
1.1 Minimal Risk
This study is minimal risk as all patients are receiving standard clinical care. This study was designed in collaboration with the Penn Medicine Musculoskeletal Service Line. The intervention is clinically appropriate and aligns with the standard patient clinical care.

1.2 Impact on Subjects Rights and Welfare
Participation in the intervention is completely voluntary. Participants randomized to the intervention arms still need to opt-in to the study. All participants receive standard clinical care.

1.3 Waiver Essential to Research
The consent process itself may influence the outcome of our study. The purpose of the HomeConnect+ program is to encourage patients to go home after surgery. The invitation to be part of HomeConnect+ includes language to nudge the patient to choose home as their discharge goal. Explaining the program to a potential participant randomized to the Usual Care arm may influence the patients decision making process for discharge disposition. It would be impractical to obtain a written signature for consent and HIPPA authorization. We are requesting a waiver of the requirement to document consent and HIPPA authorization with signature for participants enrolled in the intervention since we believe the research presents no more than minimal risk of harm to subjects and involves no procedures for which written consent is normally required outside of the research context.

1.4 Written Statement of Research
None

Potential Study Risks
As this study does not involve changes to standard patient care or medical decision making and only tests the use of remote monitoring approaches to encouraging patients to go home after surgery, we consider this study minimal risk. The primary risk would be from a breach of confidentiality involving medical records reviews and monitoring of activity tracker, or text messaging which will be maintained on the Way to Health platform. This risk has been mitigated by extensive privacy protection protocols, a highly secure data storage system, and a plan to remove identifiers from the data wherever possible. In addition, all personnel will be held to high standards of upholding confidentiality and safeguarding patient privacy.

Potential Study Benefits
The immediate benefits of this study for participants may include an improvement in recovery, both due to going home after surgery which has been proven effective in improving patient outcomes and due to feedback and milestone messaging. It is possible that the benefits for many participants will be minimal. However, as mentioned, we believe the risks are also minimal. Overall the risk to benefit ratio is favorable given the long term potential of improvement for health and health related behaviors Participants in this study may not receive any direct benefits. Some may benefit directly by going home after surgery, receiving extra support when recovering at home from the remote monitoring through the feedback and milestone messaging or feel more connected to their clinical care team. The control group

is unlikely to directly benefit, as this group will continue to simply receive usual care. Knowledge gained from the study will assist in development of interventions in other post-acute care populations. The potential public health impact of a successful intervention to improve recovery for LEJR patients is enormous and could reduce the number of readmissions and related costs in the United States each year. The risks of loss of confidentiality are minimal in this study. Thus, the benefits of this research to the participants studied, and to society at large, far surpass the risks.

Data and Safety Monitoring

The study is minimal risk to participants and therefore the Principal Investigator will monitor the safety of this study.

Risk/Benefit Assessment

Hip and knee replacements are the most common inpatient surgery for Medicare beneficiaries, with substantial cost during the hospitalization and through post-acute care, where there is substantial variability. This study is designed to test an intervention that incorporates many components that we have previously tested through rapid-cycle experimentation that we believe can improve the value and outcomes for patients who received joint replacement surgery. We believe that the combination of these approaches in this study will prove the research and public health communities with important information that can lead to broad generalizability in post-acute after for LEJR patients, as these types of interventions could be set up by healthcare system to be broadly utilized.

Summary of Protocol Changes Modifications LOG

Protocol: Automated Hovering for Joint Replacement Surgery
University of Pennsylvania Principal Investigator: Shivan Mehta, MD

Date of Submission	Description of Modification	Rationale for Modification	Approval date
1/26/18	Initial Submission		2/1/18
2/19/18	Deviation	A patient randomized to the control arm was mailed an invitation letter to join the study. A patient randomized to the intervention arm was not mailed an invitation letter to join the study. Both patients were excluded from final analyses.	2/26/18
4/2/18	Change to study documents	1. Revisions to recruitment script<br>2. Revisions to set-up letter for patients receiving study phone<br>3. Non-adherence letter for patients non-adherent for >1 week	4/5/18
4/24/18	Change to study documents<br>Update to eligibility criteria	1. Texting instructions for patients not familiar with texting<br>2. Eligibility: exclusion for non-english speaking patients	5/7/18
6/27/18	Deviation	Our protocol states that we will alert the clinical care team if a patient experiences a decrease in their average step count from last week AND an increase in their average pain score from last week. The CRC did not receive the alerts. A total of 1 alerts were missed for 1 participant due to this error.	7/6/18
7/17/18	Add study personnel	1. Catherine Reitz, Project Manager<br>2. Akriti Mishra, Data Analyst	7/23/18
7/19/18	Deviation	Our protocol states that if a participant experience 3 consecutive days of high pain score (7 or higher) the CRC will receive a message from WTH and call the participant to check-in. For participants enrolled in the Knee Goal Setting arm, the CRC was not receiving a notice for 3-day high pain score. A total of 16 alerts were missed for 3 participants due to this error. The patients received appropriate and standard clinical care and our study did remind the participants to call the Ortho hotline, which is an automated message sent to all participants with a pain score of 7 or higher, so there is no need to inform the patients. Our failure to send an in-basket message to the care team did not influence or hinder the standard clinical care the patient received. However the fact that the patient did seek clinical care at the time our study alert should have triggered, validates our data and the potential ability of our study to alert care teams to patients clinical care needs.	7/24/19

10/10/18	Change to study documents<br>Change in study procedures	1. Revisions to Nokia device set-up instructions<br>2. Change in arm 2b, patients are not docked an achievement level for not achieving step goal	10/17/19
12/19/18	Continuing Review		1/14/19
4/17/18	Clarify objectives, outcomes and analysis plan	1. Provide detailed/thorough analysis plan to be more descriptive of the specific comparisons to be made.	4/19/19
7/26/19	Add study personnel	1. Caitlin McDonald, Clinical Research Coordinator	8/5/19
8/12/19	Add study personnel	1. Erin Huang, Data Analyst	8/14/19
8/20/19	Deviation	1. Patient was randomized to the control group but received enrollment messaging for intervention group. Patient was withdrawn from study per PI discretion.	8/26/19
2/10/20	Deviation	1. Seven patients that were ineligible to participate (>85 yrs old) were erroneously randomized and enrolled in the study<br>2. Thirty-one patients named a support partner to participate along with them, with the intention they would receive messages related to non-adherence for reporting of pain scores and/or step data, as well as weekly updates on pain (if increased) and step counts (if increased or decreased) for their participants in order to provide additional support and accountability for recovery. These messages were never sent to partners; partners only received the non-adherence messages for their associated participants.	2/13/20
3/16/20	Deviation	1. Five participants identified support partners to participate along with them in the study. These partners were never contacted or added into Way to Health, and therefore never received the text messages they were supposed to.	4/3/2020

Initial Statistical Analysis Plan

Analysis Plan

1.1 Power and sample size

We expect the rate of discharge to home to be approximately 53% through usual care. With 300 patients we will have at least 80% power to detect an increase of 15% among those receiving the intervention, using a two-sided test with a significance level of 0.05. We anticipate approximately 35 patients in arms 2a and 2b. Based on a standard deviation in average daily step count of 1200 with a two sided alpha of 0.05, we have 80% power to detect an increase in average daily step count of 800 from week 2 to week 6 in arm 2b as compared to arm 2a.

1.2 Data analysis

We expect the rate of discharge to home to be approximately 53% through usual care. With 300 patients we will have at least 80% power to detect an increase of 15% among those receiving the intervention, using a two-sided test with a significance level of 0.05. We anticipate approximately 35 patients in arms 2a and 2b. Based on a standard deviation in average daily step count of 1200 with a two sided alpha of 0.05, we have 80% power to detect an increase in average daily step count of 800 from week 2 to week 6 in arm 2b as compared to arm 2a.

Final Statistical Analysis Plan

****New changes from initial protocol notated in bold, parts removed from initial protocol notated in strikethrough**

Analysis Plan

<u>1.1 Power and sample size</u>

We expect the rate of discharge to home to be approximately 53% ~~through~~ **for patients receiving** usual care. With 300 patients we will have at least 80% power to detect an increase of ~~15~~ **16**% among those receiving the intervention, using a two-sided test with a significance level of 0.05.

Among the 150 patients who are offered the intervention, w**~~W~~e anticipate approximately** 70 patients will enroll in and receive hovering. They will be randomized in a 1:1 ratio to **35 patients** each **in arms 2a and 2b. Based on a standard deviation in average daily step count of 1200 with a two sided alpha of 0.05, we have 80% power to detect an increase in average daily step count of 800 from week 2 to week 6 in arm 2b as compared to arm 2a**

<u>1.2 Data analysis</u>

We expect the rate of discharge to home to be approximately 53% ~~through~~ **for patients receiving** usual care. With 300 patients we will have at least 80% power to detect an increase of ~~15~~ **16**% among those receiving the intervention, using a two-sided test with a significance level of 0.05. **We will compare discharge status proportion using the chi-squared test of proportions.**

Among the 150 patients who are offered the intervention, w~~W~~e anticipate approximately **70 patients will enroll in and receive hovering. They will be randomized in a 1:1 ratio to** 35 patients **each** in arms 2a and 2b. Based on a standard deviation in average daily step count of 1200 with a two sided alpha of 0.05, we have 80% power to detect an increase in average daily step count of 800 from week 2 to week 6 in arm 2b as compared to arm 2a. **We will use the independent group t-test to compare the difference in average daily step count from baseline to the end of the intervention between arms 2a and 2b.**

Summary of Statistical Analysis Plan Modifications

Before beginning any analysis, we felt it necessary to revisit outcomes and provide a more detailed/thorough analysis plan to ensure both were as complete as possible. The analysis plan was updated to be more descriptive of the specific comparisons that would be made.

Appendix A: Text Messaging Content

Target	Content	Timing
Arms 2a & 2b	Welcome to HomeConnect+! Please remember to wear your Nokia pedometer daily. If you have any questions please call us at xxx-xxx-xxxx	Day of discharge
Arms 2a & 2b	Good morning from HomeConnect+ On a scale of 1-10, how would you rate your pain today? (0= no pain, 10 = worst possible pain). Please respond with only the number	Once daily starting day of discharge x 14 days
Arms 2a & 2b	Your pain score is high. If it feels unmanageable, call the Ortho hotline at xxx-xxx-xxxx	If reported pain score is >= 7
Arms 2a & 2b	Hi (Participant Name)! It has been at least 3 days since you sent us your daily pain score. Please reply with your pain level (0-10, 10=high) as soon as possible. If you are having issues call us: XXX-XXX-XXXX	If no pain score reported in the last 3 days
Arms 2a & 2b	Hi (Participant Name)! It's been at least 3 days since you uploaded your Nokia data. Please upload as soon as possible. If your having issues, call us: XXX-XXX-XXXX	If no upload in the last 3 days
Arms 2a & 2b	Hi from the HomeConnect+ team! Continue to manage pain, inflammation and swelling with pain medication, ice, and rest.	2 days post-discharge
Arms 2a & 2b (Knee Only)	Try to keep your knee straight and in an extended position. If you elevate your leg place pillows at your calves or ankles to keep your knee straight.	4 days post- discharge
Arms 2a & 2b (Hip Only)	Review the hip precautions from your surgeon and PT, it is important to know movements and activities you should avoid for 6 weeks after your surgery.	4 days post-discharge
Arms 2a & 2b (Knee Only)	Hi (Participant Name) continue to focus on getting full knee extension and increasing range of motion on a daily basis – HomeConnect+ Team .	6 days post- discharge
Arms 2a & 2b (Hip Only)	Hi (Participant Name) continue to focus on your exercises, including hip and knee exercises once a day; at week 2, change to 2-3 sets daily – HomeConnect+ Team	6 days post-discharge

Arms 2a & 2b	Try to bath and dress on your own, without physical assistance from someone else, unless you feel unsafe. It's ok to have a family member or caregiver nearby for safety.	9 days post-discharge
Arms 2a & 2b (Knee Only)	Hi (Participant Name), spend the majority of your day out of bed and walk within your home environment, at least every hour – the HomeConnect+ Team	12 days post- discharge
Arms 2a & 2b (Hip Only)	Hi (Participant Name), try to build tolerance by increasing activity by 5-10 minutes every day. - the HomeConnect+ Team	12 days post-discharge
Arms 2a & 2b	Hi (Participant Name), now is a good time to start to think about decreasing your pain medications - the HomeConnect+ Team	15 days post-discharge
Arms 2a & 2b	Walk up and down your household steps using the rail and cane for assistance one to two times daily or as tolerated – your friends at HomeConnect+	17 days post-discharge
Arms 2a & 2b	Hi (Participant Name) continue to focus on your exercises, including hip and knee exercises twice a day, 10 – 20 reps, 2 – 3 sets – HomeConnect+ Team	20 and 26 days post-discharge
Arms 2a & 2b	Hi (Participant Name), continue to try to decrease your pain medications - the HomeConnect+ Team	23 days post-discharge
Arms 2a & 2b	Hi (Participant Name), continue to ice as needed. Begin to use heat for stiffness. Decrease the use of pain medications – your friends at HomeConnect+	31 days post-discharge
Arms 2a & 2b	Hi from the HomeConnect+ team! Try to walk the length of 2-3 blocks independently. Use your walking aide as needed. Have a friend or family member around to feel more comfortable.	39 days post-discharge
Arms 2a & 2b	Hi (Participant Name) Thank you for participating in HomeConnect+ Today is your last day in the program. You may keep the Nokia activity tracker.	42 days post-discharge
Arms 2a & 2b	Congratulations on graduating from HomeConnect+! Please follow the above link to complete our short 3-question survey about your experience	45 days post-discharge
Arm 2b only	Congratulations you have reached Bronze level for your step count. Your average step count this week was XXXX, if you reach XXXX next week you can move from Bronze to Silver status!	7 days post-discharge
Arm 2b only	Hi (Participant Name) Congratulations! Your average step count this week increased from last week! You are now (level) status! If you increase your average step count to #### next week you will move to (level) status!	Starting 14 days post-discharge if step count increased >=5%, once weekly thereafter per step avg

Arm 2b only	Hi (Participant Name), congratulations! Your average step count this week increased from last week! You are at the Platinum level! Increase your average step count to #### to stay on top!	After Platinum status achieved, per step avg
Arm 2b only	Hi (Participant Name) Congratulations! Your average step count this week increased from last week! Keep up the good work and you can move to the next level next week!	Starting 14 days post-discharge if step avg increased less than 5% over past week, once weekly thereafter per step avg
Arm 2b only	Hi (Participant Name), we've noticed that your average step count this week is less than last week, we will be calling you shortly to check-in and see if everything is okay.	Starting 14 days post-discharge if step avg decreased, once weekly thereafter per step avg
Partners (2b)	Welcome to HomeConnect+ ! We will be sending you progress updates about your friend PARTICIPANT_FIRSTNAME. If you have any questions please call us at xxx-xxx-xxxx.	Day of discharge
Partners (2b)	Your friend (Participant Name), achieved their weekly goal this week for step count and have moved up to (level) status! Congratulate them and encourage them to keep up the good work! –HomeConnect	Never sent
Partners (2b)	Your friend (Participant Name), average step count this week is higher than last week. Send you congratulations and encourage them to keep up the good work!	Never sent
Partners (2b)	(Participant Name) average step count this week is less than last week. Please check-in and see if they need any assistance with their recovery.	Never sent
Partners (2b)	(Participant Name) average pain score this week is more than last week. Please check-in and see if they need any assistance with their recovery.	Never sent
Partners (2b)	Hi, it's been at least 4 days since (Participant Name) uploaded their Nokia daily step data. Please encourage them to do so as soon as possible.	If no upload in the past 4 days
Partners (2b)	Hi, it's been at least 4 days since (Participant Name) has sent us their daily pain score. Please encourage them to do so as soon as possible.	If no pain score reported in past 4 days

Appendix B: End of Study Final Survey Instrument (Arms 2a & 2b only)

1) On a scale of 1-10, how likely are you to recommend the HomeConnect+ program to someone undergoing hip or knee replacement? (1= extremely unlikely, 10= extremely likely)

2) On a scale of 1-5, how do you agree with this statement? 'The HomeConnect+ program made me feel more connected to my care team.' (1= strongly disagree, 2= disagree, 3= neutral, 4= agree, 5= strongly agree)

3) On a scale of 1-5, how do you agree with this statement? 'The HomeConnect+ program made me feel more comfortable going home.' (1= strongly disagree, 2= disagree, 3= neutral, 4= agree, 5= strongly agree)
