# Supplementary material for: Effect of Remote Monitoring on Discharge to Home, Return to Activity, and Rehospitalization After Hip and Knee Arthroplasty: A Randomized Clinical Trial
Source: JAMA Netw Open. 2020 Dec 21;3(12):e2028328. doi: 10.1001/jamanetworkopen.2020.28328 (PMC7753899; doi:10.1001/jamanetworkopen.2020.28328)
Supplement: Supplement 2. — eTable 1. Timed Up and Go (TUG) Scores eTable 2. Device Usage by Study Arm (n=96, All pts Who Opted to Receive Device, Weeks 1-6) eFigure. Intervention Flow Diagram [file jamanetwopen-e2028328-s002.pdf]

Supplemental Online Content

Mehta SJ, Hume E, Troxel AB, et al. Effect of remote monitoring on discharge to home, return to activity, and rehospitalization after hip and knee arthroplasty: a randomized clinical trial. *JAMA Netw Open*. 2020;3(12):e2028328.
doi:10.1001/jamanetworkopen.2020.28328

eTable 1. Timed Up and Go (TUG) Scores
eTable 2. Device Usage by Study Arm (n=96, All pts Who Opted to Receive Device, Weeks 1-6)
eFigure. Intervention Flow Diagram

This supplemental material has been provided by the authors to give readers additional information about their work.

© 2020 Mehta SJ et al. *JAMA Network Open*.

eTable 1. Timed Up and Go (TUG) Scores

	**Intervention**	**Control**	**P-value**
	**N=118**	**N=123[5]**	
**TUG Scores Available[1]** (%)	67 (56.8)	71 (57.7)	
First TUG Score (seconds), Median (IQR)	36 (28 – 49)	38 (26 - 52)	0.99
Final TUG Score (seconds), Median (IQR)	15 (11 – 18)	15 (11 – 17.5)	0.81
**Change in TUG per day (sec/day),** Median (IQR)	-1.3 (0.6 – 2.3)	-1.2 (0.5 – 2)	0.61
**Absolute Difference in TUG,** Median (IQR)	20 (12 – 34)	18 (12 – 37)	0.85

[1] Patients with at least 2 TUG scores, TUG scores coded as 0 were censored

© 2020 Mehta SJ et al. *JAMA Network Open*.

eTable 2. Device Usage by Study Arm (n=96, all pts who opted to receive device, weeks 1-6)

**Device Usage**	**Study Arm**		**P-value**
	**2a N=44**	**2b N=52**	
# Days, Median (IQR)	30 (0-39)	33 (12-41.5)	.21
# Weeks, Median (IQR)	6 (0-6)	6 (3-6)	.36
# Days, Mean (SD)	21.7 (17.9)	26.3 (15.8)	.18
# Weeks, Mean (SD)	3.7 (2.7)	4.3 (2.3)	.21

© 2020 Mehta SJ et al. *JAMA Network Open*.

eFigure. Intervention flow diagram

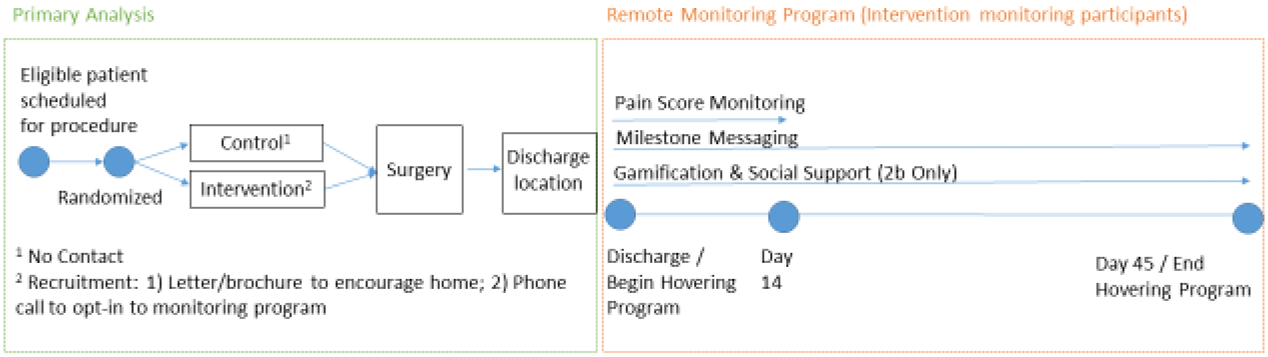

© 2020 Mehta SJ et al. *JAMA Network Open*.
