# Supplementary material for: Effect of Remote Monitoring on Discharge to Home, Return to Activity, and Rehospitalization After Hip and Knee Arthroplasty: A Randomized Clinical Trial
Source: JAMA Netw Open. 2020 Dec 21;3(12):e2028328. doi: 10.1001/jamanetworkopen.2020.28328 (PMC7753899; doi:10.1001/jamanetworkopen.2020.28328)
Supplement: Supplement 3. — Data Sharing Statement [file jamanetwopen-e2028328-s003.pdf]

Data Sharing Statement

Mehta. Effect of Remote Monitoring on Discharge to Home, Return to Activity, and Rehospitalization After Hip and Knee Arthroplasty. *JAMA Netw Open*. Published December 21, 2020.
doi:10.1001/jamanetworkopen.2020.28328

Data
Data available: No
